# The Morphoregulatory Role of Thidiazuron: Metabolomics-Guided Hypothesis Generation for Mechanisms of Activity

**DOI:** 10.3390/biom10091253

**Published:** 2020-08-28

**Authors:** Lauren A. E. Erland, Ryland T. Giebelhaus, Jerrin M. R. Victor, Susan J. Murch, Praveen K. Saxena

**Affiliations:** 1 Department of Chemistry, University of British Columbia, Kelowna, BC V1V 1V7, Canada; lauren.erland@ubc.ca (L.A.E.E.); ryland.giebelhaus@ubc.ca (R.T.G.); 2 Department of Plant Agriculture, University of Guelph, Guelph, ON N1G 2W1, Canada; jvictor@uoguelph.ca (J.M.R.V.); psaxena@uoguelph.ca (P.K.S.)

**Keywords:** thidiazuron, phytohormone, metabolomics, plant growth regulator, plant growth and development, morphongenesis, mechanism of action, herbicide

## Abstract

Thidiazuron (TDZ) is a diphenylurea synthetic herbicide and plant growth regulator used to defoliate cotton crops and to induce regeneration of recalcitrant species in plant tissue culture. In vitro cultures of African violet thin petiole sections are an ideal model system for studies of TDZ-induced morphogenesis. TDZ induces de novo shoot organogenesis at low concentrations and somatic embryogenesis at higher concentrations of exposure. We used an untargeted metabolomics approach to identify metabolites in control and TDZ-treated tissues. Statistical analysis including metabolite clustering, pattern and pathway tools, logical algorithms, synthetic biotransformations and hormonomics identified TDZ-induced changes in metabolism. A total of 18,602 putative metabolites with extracted masses and predicted formulae were identified with 1412 features that were found only in TDZ-treated tissues and 312 that increased in response to TDZ. The monomer of TDZ was not detected intact in the tissues but putative oligomers were found in the database and we hypothesize that these may form by a Diels–Alder reaction. Accumulation oligomers in the tissue may act as a reservoir, slowly releasing the active TDZ monomer over time. Cleavage of the amide bridge released TDZ-metabolites into the tissues including organic nitrogen and sulfur containing compounds. Metabolomics data analysis generated six novel hypotheses that can be summarized as an overall increase in uptake of sugars from the culture media, increase in primary metabolism, redirection of terpene metabolism and mediation of stress metabolism via indoleamine and phenylpropanoid metabolism. Further research into the specific mechanisms hypothesized is likely to unravel the mode of action of TDZ and to provide new insights into the control of plant morphogenesis.

## 1. Introduction

**Figure 1:**
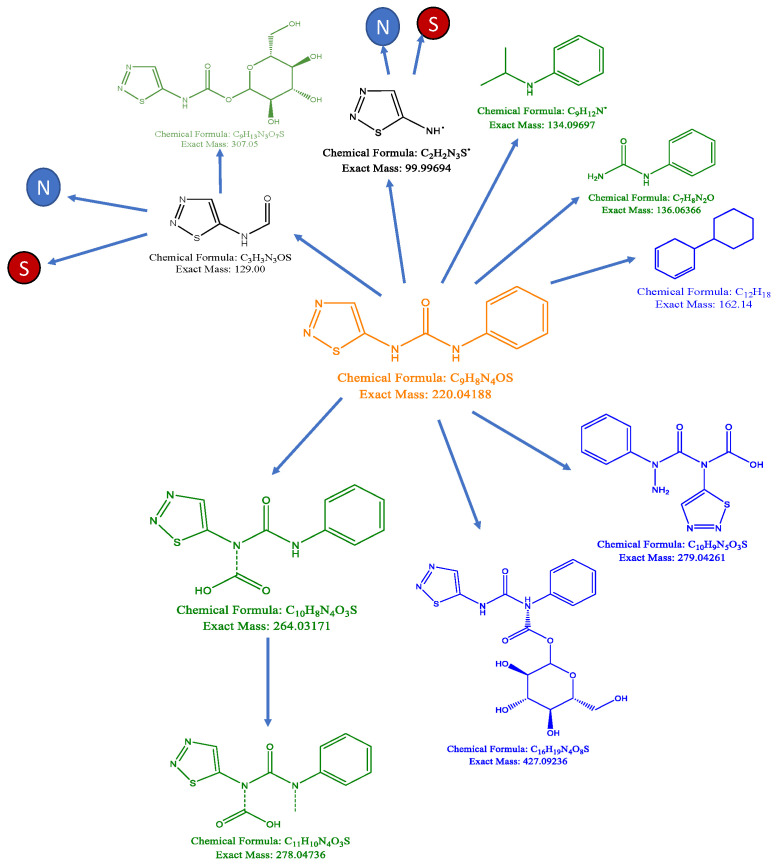
Thidiazuron (TDZ) metabolism in plant tissues. Orange is the TDZ parent. Blue represents features found in both low and high TDZ treatments. Green represents features found only in the low TDZ treatment. Black compounds are predicted but were not detected in the dataset. Dashed lines indicate predicted bonds consistent with the molecular features.

Thidiazuron (TDZ; N-phenyl-N′-l,2,3-thidiazol-5-ylurea) is an *N,N*′-diphenylurea derivative that was originally developed as a cotton defoliant (SN 49537; Figure 1) [1,2]. Products containing TDZ alone or in combination with ethephon, diuron, glyphosate or protoporphyrinogen-(IX) oxidase (PPO) inhibitors are widely used in modern agriculture. The commercial products Adios (Arysta LifeScience), FreeFall SC (NuFarm Americas Inc.) and others containing TDZ are used to induce senescence of cotton leaves allowing for the large-scale harvest of cotton. Proposed mechanisms of action include crosstalk with cytokinins [3] or increased levels of ethylene relative to auxin in leaf petioles activating the abscission zone to defoliate the plants [4]. Some cotton cultivars are more sensitive than others [3,5,6]. In other crops, delayed senescence and increased shelf life have been reported via alternate mechanisms [7,8]. TDZ has also been found to have diverse effects in fruit crops. For example TDZ has been found to improve fruit size in kiwi (*Actinidia deliciosa* ‘Hayward’), pear (*Pyrus communis* L. cv ‘Spadona’ and ‘Coscia’) and grape (*Vitis vinifera* cv ’Simone’) [9,10,11], to increase fruit set in apple (*Malus domestica* cv ‘Fuji’, ‘Gala and MacIntosh) and pear (*Pyrus calleryana* cv ‘Hosui’ and ‘Packham’s Triumph’ [12,13,14,15], and increase yield in pear (*P. calleryana* cv ‘Hosui’ and ‘Packham’s Triumph’) and cucumber (*Cucumis sativa* L.) [15,16].

TDZ is one of the most widely used plant growth regulators for induction of de novo regeneration, shoot organogenesis, somatic embryogenesis and callus in hundreds of species, spanning horticultural, ornamental, medicinal, woody and crop plants [2,17,18,19]. An average of 86 new reports of TDZ-induced in vitro regeneration have been published each year since 2010 (Web of Science, 2020). In tissue culture systems, it has been proposed that TDZ acts through the adenine-type cytokinin activity either by stimulating endogenous cytokinins or binding to cytokinin receptors [1,2,7,18,20,21,22,23]; however, the cytokinin model does not adequately explain the diversity of physiological responses in different species [2,24,25,26]. In some systems, application of TDZ more closely mimics the application of exogenous auxins [24,25,27,28] and stimulation of endogenous auxin metabolism has been proposed [24,29]. Studies of endogenous plant growth regulators under TDZ application hint at a complex system of phytohormone crosstalk directing morphogenesis [3,25,27,30,31]. Interestingly, TDZ responses are also dependent on dose, exposure time, light and other environmental cues [18,28,30,32]. Despite decades of research and widespread commercial application, the specific mode(s) of action of TDZ in plant growth regulation remain undefined.

TDZ-induced regeneration in African violets (*Saintpaulia ionantha*, CV. Benjamin) is an ideal model system for studies of the mechanism of action of TDZ [29,32,33,34]. Regeneration proceeds through distinct physiological pathways dependent on the level of exposure to TDZ [29,32]. At low levels of TDZ, African violet petioles produce de novo shoots and at higher levels of exposure, petioles form somatic embryo-like structures (Supplementary Figure S1) [29,32]. The timing of exposure of African violet tissues to TDZ was not critical to the overall regeneration outcome [29], but the morphogenic response was found to be dependent on the intercellular transport of auxin and calcium [29].

The current study was undertaken to understand the morphoregulatory role of TDZ in African violet. A metabolomics-based approach was used to generate an untargeted dataset representing significant changes in metabolites [35,36]. Metabolomics is the term that describes the comprehensive, non-biased, high-throughput, state-of-the-art mass spectroscopic analysis of the whole profile of metabolites in a complex system such as a plant cell [35,36]. We hypothesized that TDZ initiates changes in the metabolome of African violet petiole sections to redirect plant growth. Our approach suggests six novel hypotheses for future research programs to understand the mode of action of TDZ in plant cells. Understanding the morphoregulatory role of TDZ will provide new insights into plant regeneration and control of plant growth and development in vitro.

## 2. Materials and Methods

### 2.1. In Vitro Grown Plant Tissues

In vitro stock plants of African violet (*Saintpaulia ionantha*, CV. Benjamin) were established in perpetual axenic culture as described previously [32,37,38]. Petiole explants were excised and cultured on a medium containing MS salts and vitamins (Murashige and Skoog 1962), 3% sucrose and 0, 2 or 20 μM thidiazuron (TDZ; Sigma, Mississauga, ON, Canada) with pH adjusted to 5.7 and solidified with 2.5% gellan gum (Schweitzerhall, South Plainfield, NJ, USA). Media were autoclaved at 121 °C for 20 min. The experiment was designed with control, low TDZ and high TDZ treatments using petiole cross-sections excised from the in vitro grown maternal stocks. Control explants were cultured on basal MS medium devoid of exogenous growth regulators for six days. Low TDZ-treated explants were cultured on the 2 μM TDZ medium for three days. High TDZ-treated explants were cultured on the 20 μM TDZ medium for six days. All cultures were incubated in a controlled environment growth room at 24 °C in light (16/8-h (day/night) photoperiod; 50 μmol s^−1^ m^−2^) provided by cool-white fluorescent lamps (Philips Canada, Scarborough, ON, Canada) [29,32]. Resulting growth is shown in Supplementary Figure S1.

### 2.2. Metabolomics Analyses

TDZ-induced petiole sections were harvested from the cultures, immediately flash frozen in liquid nitrogen, stored at −80 °C and couriered overnight on dry ice to the facility for untargeted metabolomics (Phenomenome, Saskatoon, SK, Canada). Detailed standard operating methods have been published previously [39,40,41]. In brief, 300 mg of each sample was accurately weighed and metabolites were separated into extracts of 3 mL of either methanol/0.1% formic acid (50%/50%) or 100% acetonitrile solutions. Extracts were sonicated ≈15 min, filtered (0.2 mm PTFE filter), and diluted 1:19 prior to ESI and APCI analysis. Mobile phases (a) 50/50 MeOH/0.1% ammonium hydroxide and (b) 50/50 MeOH/0.1% formic acid were used for dilution and elution of all negative and positive ion ionization analysis to create a series of extracts of each sample with differing polarities and acid/base chemistries (Supplementary Table S1) [41,42].

#### 2.2.1. FTMS Analysis

Extracts were analyzed with Fourier Transform Ion Cyclotron Mass Spectrometer (FTMS; Bruker Daltonics APEX III) equipped with a 7.0-Tesla actively shielded superconducting magnet and ESI and APCI sources. ESI, APCI, and ion transfer conditions were optimized using a standard mix of serine, tetra-alanine, reserpine, Hewlett-Packard tuning mix, and the adrenocorticotrophic hormone fragment 4–10. Instrument conditions were optimized for ion intensity and broadband accumulation and calibrated for mass accuracy over the range of 100–1000. Data were subjected to Fourier transformation and magnitude calculations as previously described [39]. All mass deviances from calibration standard curves were 1.0 ppm over the mass range studied [39]. Extraction efficiency was tracked with the same standards and multiple, independent FTMS analyses were performed on each extract [39].

#### 2.2.2. FTMS Data Processing

The mass spectra from each analysis were integrated following calibration, creating a peak list that contained the exact mass and absolute intensity of each peak as previously described [39,41]. This raw peak list was filtered to remove all ^13^C isotopes and deconvolution analysis was performed on each of the peaks in the filtered peak list to predict molecular formulae consisting of carbon, hydrogen, nitrogen, oxygen, phosphorus, and sulfur, with a single charge and with an error of <1.0 ppm [39]. Features that met these criteria along with the proposed empirical formulae and error of determination were stored in the final processed data file. In order to compare and summarize data across different ionization modes, detected mass peaks were converted to their corresponding neutral masses and used to sort the data accordingly [39].

#### 2.2.3. Data Processing and Multivariate Statistics

Data from all replicated analysis of all extracts were compiled into a single array using the proprietary Phenomenome software [41] and putative empirical formulae were assigned out on the basis of accurate mass data (mass accuracies <1 ppm). For data analysis requiring *m*/*z*, data were aligned and processed in Excel™. Data were then submitted to MetaboAnalyst (v4.0) [42,43,44,45] and scaled by mean centering and subjected to multivariate statistics (principal component analysis; PCA and partial least squares-discriminant analysis (PLS-DA). As no clear delineation of groups was provided by multivariate analysis the following further analyses were undertaken.

#### 2.2.4. Detection of Significant Ions and Linear Trends

Significant features were putatively identified in Excel and matched by monoisotopic mass in FooDB (±0.02 Da) [46]. Those which did not yield a plausible match were transformed through the loss of one (−220.041882) or two (2 × −220.041882) TDZ molecules to identify potential conjugates. Data submitted to MetaboAnalyst were subjected to significant analysis of microarray (SAM) analysis to identify possible important features. Pattern Hunter was additionally applied to identify features which showed a linear increase (control < 2 < 20), decrease (control > 2 > 20), or a peak or dip at 2 µM TDZ with Pearson r used as the distance measure. Only positive correlations were retained for further analysis to allow for differentiation between increasing and decreasing patterns.

Pairwise analysis between control and high TDZ and control and low TDZ were additionally performed in MetaboAnalyst by t-test, volcano analysis, significant analysis of microarray (SAM) analysis and empirical Bayes analysis of microarrays (EBAM) to identify possible important features in specific morphogenetic responses (organogenesis at low TDZ and embryogenesis at high TDZ) [43,44,46,47].

#### 2.2.5. Synthetic Biotransformations

**Figure 2:**
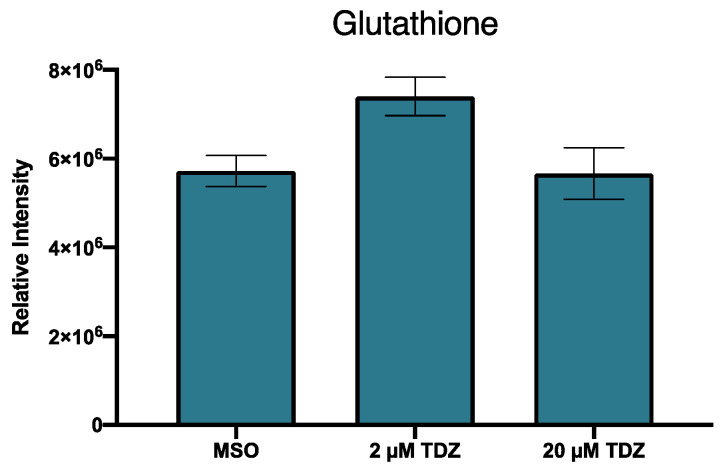
Relative expression of metabolite putatively identified as glutahione in African violet petioles treated with 0, 2 or 20 µM thidiazuron. Bars represent mean, with error bars represent standard error margins.

**Figure 3:**
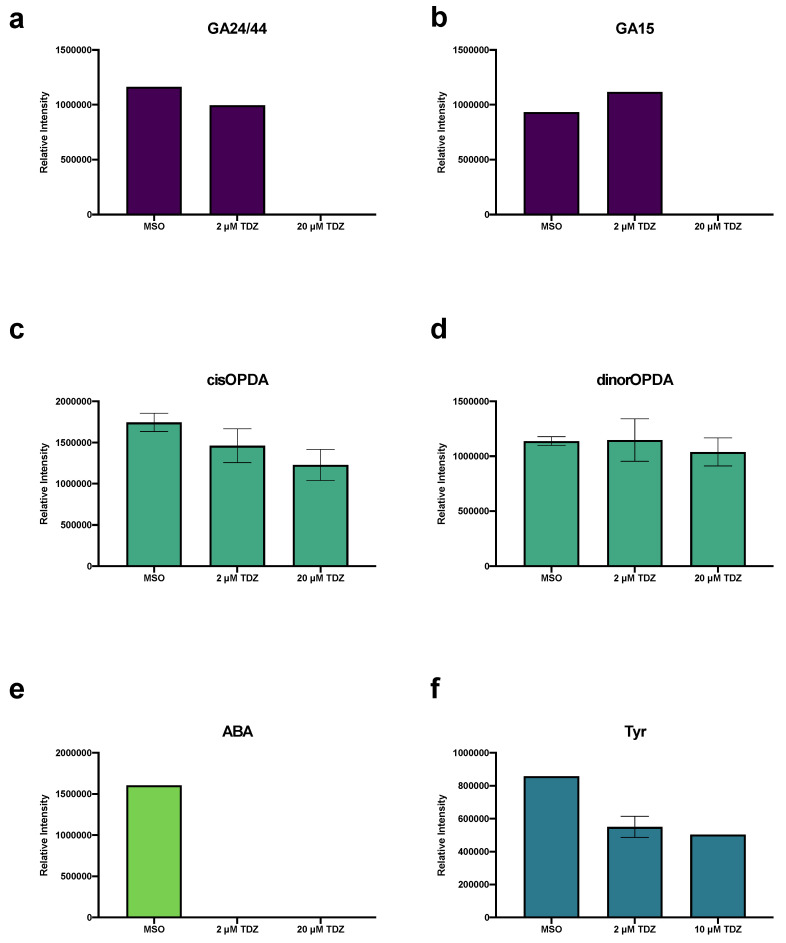
Effects of thidiazuron (TDZ) treatment on gibberellins (GA; (**a**,**b**)), jasmonates (12-oxo-phytodienoic acid; OPDA; (**c**,**d**)), abscisic acid (ABA; (**e**)) and tyrosine (Tyr; (**f**)) levels, identified by hormonomics analysis in African violet petioles. Bars represent mean, error bars span standard error margin. Where no error bars are displayed, the feature was present in only one replicate.

Using previously described methods synthetic biotransformations were applied to the thidiazuron molecule in Excel: −H_2_, +H_2_, −CH_3_, +CH_3_, +C_6_H_12_O_6_, −C_6_H_12_O_6_, +OH, +OH (×2), +COOH, +NH_2_, +NH_3_, −H, +H [37,47,48,49]. The monoisotopic mass for each biotransformation (±0.02 Da) was mined within our dataset in order to identify potential anabolic or metabolic products. Additionally, monoisotopic masses for predicted dimers and trimers as previously predicted [2], and breakdown products (Figure 2 and Figure 3) were mined within the dataset.

#### 2.2.6. Pathway Analysis

The results from pairwise t-tests and volcano analysis along with pattern searching were input in Peaks to Pathways (ranked by *p*-value for volcano analysis, formatted as three column m.z, *p*-value, t.score for all other results), with mass accuracy (ppm) of 5, in positive mode and subjected to Mummichog (*p*-value cut off set to top 10 percent of peaks) and gene set enrichment analysis (GSEA) pathway analysis, with mapping to known *Arabidopsis thaliana* KEGG metabolic pathways (v. October 2019), using standard settings for adducts and currency metabolites [46]. Data were compared to known carbon metabolism pathways (KEGG v 2019) by matching to monoisotopic mass (±0.02 Da).

#### 2.2.7. Hormonomics Analysis

A hormonomics analysis was also performed by mining the monoisotopic mass for phytohormones and their related metabolites within the dataset (±0.02 Da) [46]. The query metabolites are summarized in Supplementary Table S2. Metabolites putatively identified in the hormonomics analysis were compared by analysis of variance (ANOVA) with Tukey’s honestly significant difference multiple comparisons model. Samples in which the analyte was not detected were replaced with a very small number for the purposes of ANOVA analysis only and were left blank for preparation of bar charts. Significance was set at α = 0.05.

#### 2.2.8. Putative Compound ID

Significant features identified through MetaboAnalyst analysis and features predicted through biotransformations or logical algorithms were uploaded into FooDB (M + H, v1.0, foodb.ca, ±0.02) and ChemSpider (M ± 0.001 Da) for putative identification. All identifications are putative and are based on *m*/*z* [36,39,41,42,46]. In all cases where a feature was detected in multiple modes the relative peak intensities were summed prior to plotting.

## 3. Results

### 3.1. Database Compilation and Multivariate Analysis

African violet data was compiled into an array encompassing 18,602 putative metabolites with extracted masses and predicted formulae (Supplementary Table S3). About 2200 metabolites were common to all samples representing primary metabolites required for cell viability (Supplementary Table S3). 1412 features were identified which were present only in TDZ-treated tissues and were not present in African violet petioles grown on basal medium, 312 of these were found to increase with TDZ treatment (Supplementary Table S3). Neither PCA nor PLS-DA identified a significant difference in metabolite levels in response to increasing TDZ treatment, due to limited replicate numbers. Percent cumulative variance from PCA was 43.9%, 27.5% and 12.8% for principle components (PC) 1–3, respectively (Supplementary Figure S2). PLS-DA had and R^2^, Q^2^ and accuracy of 1 with 4 components (Supplementary Figure S3) and percent cumulative variance explained by the model was 36.1%, 24.9% and 19% for components 1–3 respectively. Generally important features identified by Variable Importance in Projection (VIP) scores are those with the highest peak intensities. Classes of compounds putatively identified as important features through this analysis include terpenoids, dipeptides, flavonoids, anthraquinones, coumarins and ethanylamines (Table 1).

### 3.2. Logical Algorithms

To identify features associated with somatic embryogenesis and general TDZ treatment data were mined to identify features which were absent in MSO and increased with exposure. Twelve metabolites were putatively identified that were below detection limits in control tissues and increased with increasing TDZ treatment including terpenes, polyphenols, fatty acids, and other small molecules (Table 2). While several of the features show linear trends, the signal putatively identified as hyperforin has an almost perfect linear relationship with TDZ treatment with an R^2^ of >0.99 (Supplementary Figure S4). Eight metabolites were identified that were below detection limits in control tissues and at highest signal intensity at 2 µM TDZ and included metabolite classes: fatty acids, flavones and other polyphenols, ascorbate, betalain, diacylglycercol and terpenes (Table 2). Those masses which did not have a match in FooDB were carried forward to mine for potential TDZ or 2 X TDZ conjugates and identified several putative classes of metabolites which may be conjugated to or represent chemical modification of intact TDZ or TDZ dimers in plant tissues. Classes which were found to be increased at 20 µM TDZ were: dinitrotoluene, oxygenation, phenolics, magnesium and phospholipids, those which were accumulated at the highest levels at 2 µM included: tetrahydrofolic acid, dipeptides, phospholipids, and ketones (Table 3).

### 3.3. Synthetic Biotransformations

An exact match for TDZ (Chemical Formula: C_9_H_8_N_4_O_S_; Exact Mass: 220.04188; *m*/*z*: 220.04188 (100.0%), 221.04524 (9.7%), 222.03768 (4.5%), 221.03892 (1.5%) was not detected in any of the samples. Therefore, the dataset was mined for the presence of predicted TDZ breakdown products as well as the presence of TDZ oligomers in the dataset. Breaking of the amide bridge of the TDZ molecule was predicted to be the most likely degradation pathway for the TDZ molecule (Figure 1). Carboxylation, methylation and amination of the intact TDZ molecule were also predicted (Figure 1). In addition to the likely presence of TDZ dimer conjugates (Table 3) masses consistent with the formation of TDZ trimers were identified in TDZ-treated tissues (Supplementary Figure S5).

### 3.4. Pairwise Analysis

Statistical tools to identify features or pathways which were associated specifically with organogenesis (2 µM TDZ) or embryogenesis (20 µM TDZ) identified steroid biosynthesis, folate biosynthesis, histidine biosynthesis purine biosynthesis and starch and sucrose metabolism as pathways important to organogenesis (Table 4), along with several saponins, pangamic acid, glucosides, triglycerides, one indole derivative (1-Methyl-3-(2-thiazolyl)-1H-indole) and chlorogenoquinone (Table 4). No significant features were identified by SAM or EBAM analysis for 20 µM TDZ treatment (embryogenesis-related), but several features were identified as modified in response to 2 µM TDZ associated with the following metabolic pathways: steroid biosynthesis, sesquiterpene and triterpene biosynthesis, glycolysis/gluconeogenesis, pentose phosphate pathway, fructose and mannose metabolism, galactose metabolism and fatty acid degradation (Table 5).

### 3.5. Pathway Analysis

Peaks to pathways analysis of pattern searching results identified metabolite profiles which were significantly modified by TDZ treatment. Several were found to be significantly modified both in the increasing and decreasing patterns (Table 4) and included glycolysis/gluconeogenesis, fructose and mannose metabolism, amino sugar and nucleotide sugar metabolism, inositol phosphate metabolism, phosphatidylinositol signaling, and galactose metabolism. Metabolite profiles which showed increased metabolism and/or accumulation included: N-glycan biosynthesis and arachidonic acid metabolism, while those which showed reduced accumulation or potentially increase catabolism included: porphyrin and chlorophyll metabolism, starch and sucrose metabolism, pentose phosphate metabolism, valine leucine isoleucine biosynthesis, ascorbate and aldarate metabolism, caffeine metabolism, phenylalanine tyrosine and tryptophan biosynthesis, flavonoid biosynthesis and pentose and glucoronate interconversions (Table 4). Treatment with 2 µM TDZ showed a unique metabolic profile in some cases with specifically porphyrin and chlorophyll metabolism showing increased metabolism, while the photosynthetic accessory pigments, the carotenoids had significant reductions in metabolism with 2 µM TDZ treatment (Table 4). Galactose and flavonoid metabolism both reduced metabolism at 2 µM but returned to higher levels again at 20 µM TDZ treatment (Table 4). Both tyrosine metabolism and tropane, piperidine and pyridine alkaloid biosynthesis were found to have the greatest rates of metabolite accumulation at 2 µM TDZ (Table 4). Interestingly, examination of tyrosine accumulation showed that the amino acid itself was reduced with TDZ treatment, suggesting that it may be feeding into increased metabolism downstream in the pathway, though this was not found to be statistically significant (Figure 3f).

Due to identified changes in features identified as related to sugar metabolism, a targeted analysis of sugar metabolites associated with glycolysis and the pentose phosphate cycle (PPP) were mined specifically within the dataset and led to identification of several sugars which showed differential metabolism in response to TDZ treatment (Table 6). Salicin phosphate accumulated with increasing TDZ treatment but several of the sugars including the 6C sugar isomers and their phosphates were decreased by TDZ treatment (Table 6). Treatment with TDZ lead to an increase in metabolism of six carbon sugars, while metabolism of five carbon sugars and their phosphates as well as six carbon sugar phosphates all appeared to have decreased (Table 6). Several downstream products of sulphur metabolism were also suggested as potential metabolites including reduced glutathione (Figure 2), though glutathione metabolism itself appeared to be reduced based on metabolism levels of three putatively identified metabolites methionyl-glutamate, glutamylmethionine and N-gamma-L-glutanmyl-L-methionine (Table 1). The compound putatively identified as reduced glutathione was found in the greatest abundance in tissues treated with 2 µM TDZ with levels close to those observed in the control in tissues treated with 20 µM TDZ (Figure 2).

### 3.6. Hormonomics Analysis

**Figure 4:**
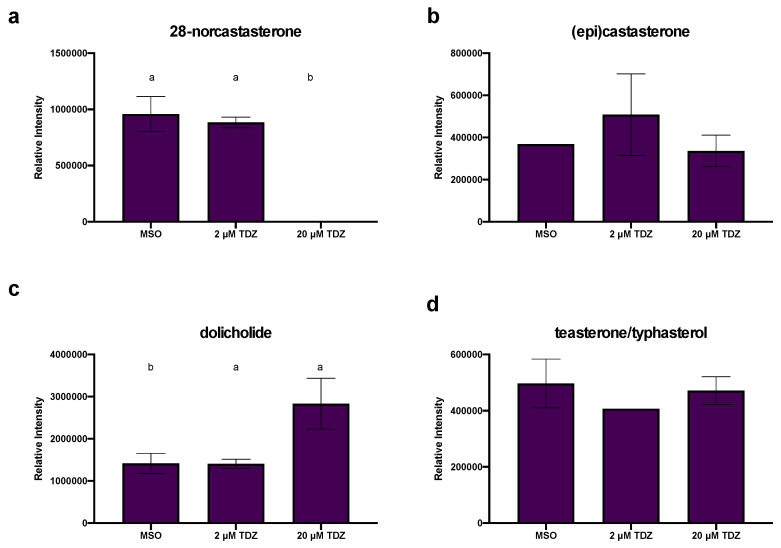
Effects of thidiazuron (TDZ) exposure on brassinosteroids (**a**) 28-norcastasterone, (**b**) epicastasterone or castasterone, (**c**) dolicholide and (**d**) teasterone or typhasterol levels in African violet petioles. Bars represent mean, error bars span standard error margin. Where no error bars are displayed, the feature was present in only one replicate.

Hormonomics analysis queried all known/predicted plant growth regulators and their major metabolites and conjugates (Supplementary Table S2) and identified features which represented several families of plant growth regulators. Gibberellins (GA) 24 and 44 (Figure 3a) and GA15 (Figure 3b) were found to be present and at relatively stable levels in MSO and 2 µM TDZ treatment but are absent at 20 µM TDZ. The jasmonate precursors cis- and dinor-12-oxo-phytodienoic acid (OPDA; Figure 3c,d) were found in all treatments with the cis version showing a linear decrease with increasing TDZ treatment, while dinorOPDA was stable. Abscisic acid (ABA, Figure 3e) was inhibited by all TDZ treatment levels. All the brassinosteroids identified in the dataset were present in all three treatment groups with the exception of 28-norcastasterone was found to be present in both MSO and 2 µM TDZ treatments but absent at 20 µM (Figure 4a). Both the precursors epicastasterone/castasterone (Figure 4b) and teasterone/typhasterol (Figure 4d) were relatively stable in all treatments while the active brassinosteroid dolicholide (Figure 4c) increased at 20 µM.

**Figure 5:**
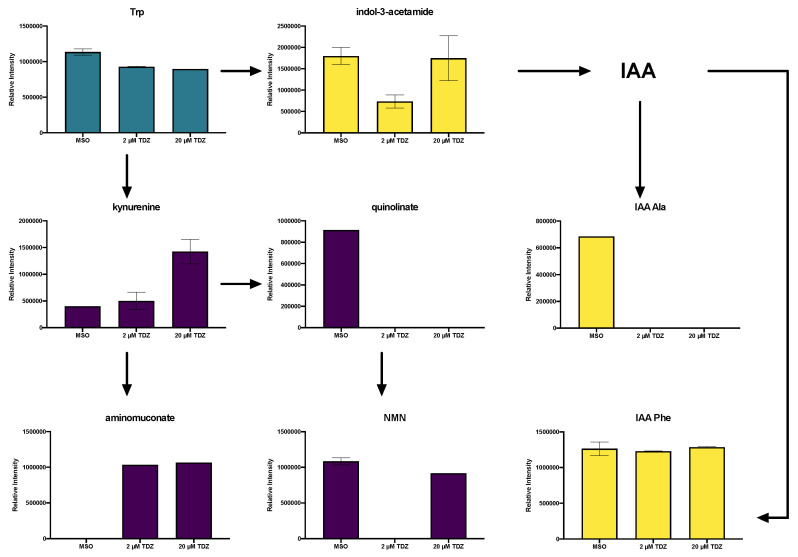
Effects of thidiazuron (TDZ) exposure on tryptophan (Trp) and auxin (inole-3-acetic acid; IAA) metabolism in African violet petioles. NMN, nicotinic acid mononucleotide. Bars represent mean, error bars span standard error margin. Where no error bars are displayed, the feature was present in only one replicate. Arrows indicate the normal flow of the pathways. Yellow indicates the IAA biosynthetic pathway, purple Trp degradation pathways.

**Figure 6:**
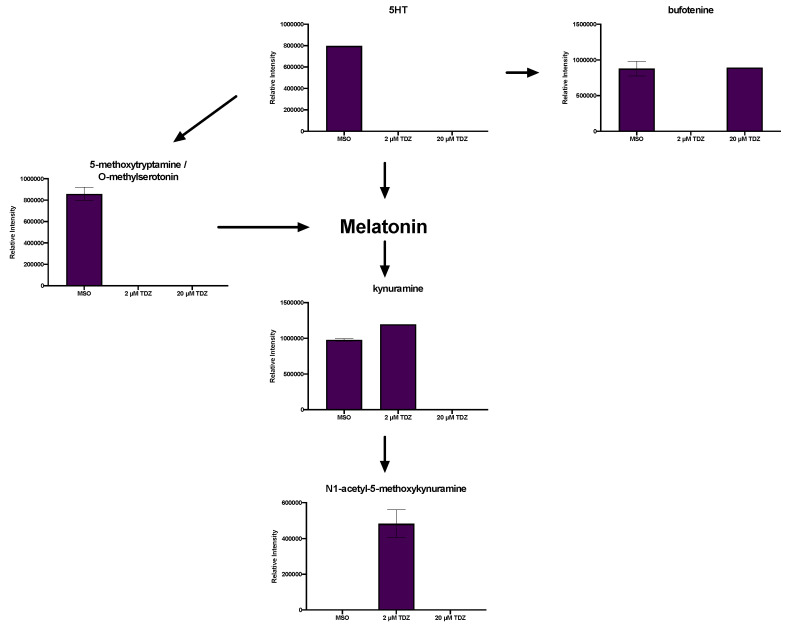
Effects of thidiazuron (TDZ) exposure on indoleamine biosynthesis and metabolism in African violet petioles. Bars represent mean, error bars span standard error margin. Where no error bars are displayed, the feature was present in only one replicate. Arrows indicate the normal flow of the pathways.

**Figure 7:**
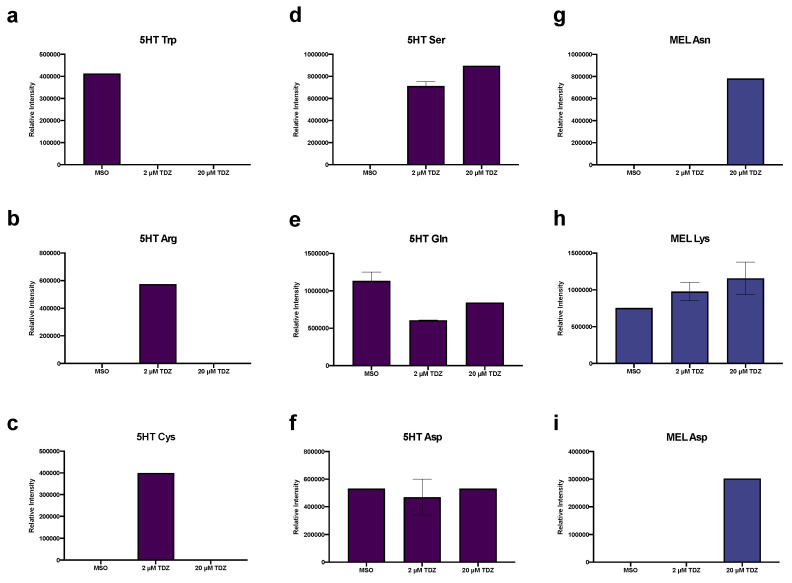
Effects of thidiazuron (TDZ) exposure on proposed indoleamine (serotonin, 5HT and melatonin, MEL) amino acid conjugates in African violet petioles. Purple denotes 5HT conjugates (**a**–**f**), blue MEL conjugates (**g**–**i**). Bars represent mean, error bars span standard error margin. Where no error bars are displayed, the feature was present in only one replicate.

Neither melatonin (MEL) nor auxin (indole-3-acetic acid; IAA) were detected in the hormonomics dataset, though putative precursors (Figure 5 and Figure 6), metabolites (Figure 5) and conjugates (Figure 5, Figure 6 and Figure 7) of these metabolites were detected. The amino acid tryptophan (Trp) was detected in all treatments, with highest levels in the MSO control (Figure 5, blue). Several metabolites in the kynurenine pathway for tryptophan degradation were identified including kynurenine itself which increased with TDZ treatment, along with aminomuconate (Figure 5, purple). Quinolinate, an alternate branch point and precursor for entry into nicotinamide metabolism, was not detected after TDZ treatment while nicotinic acid mononucleotide (NMN) was present in control and one replicate of 20 µM TDZ treatment (Figure 5, purple). The auxin precursor indole-3-acetamide was detected in all treatments as well with lowest levels observed with 2 µM TDZ treatment while the only IAA-amino acid conjugates were detected IAA-alanine (Ala) and IAA-Phenylalanine (Phe) with the former being detected only in the control and the latter present in all replicates of all treatments at very stable levels (Figure 5, yellow).

**Figure 8:**
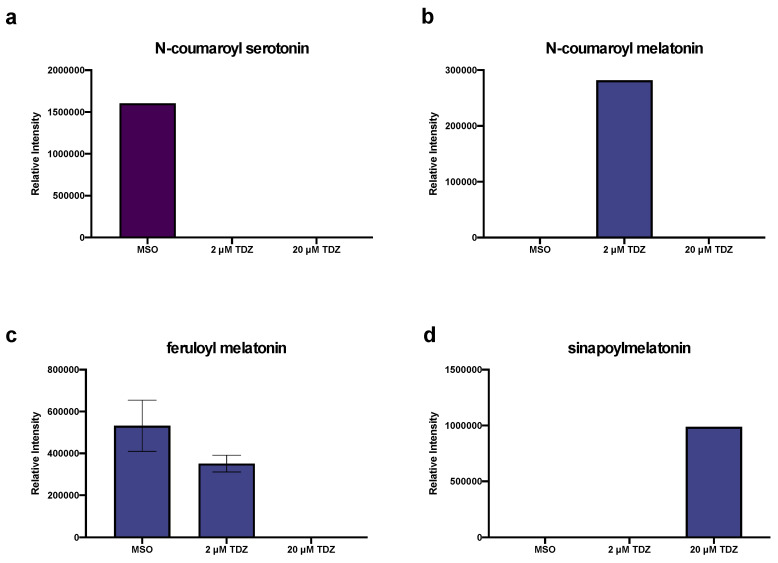
Effects of thidiazuron (TDZ) exposure on proposed indoleamine (serotonin (5HT) and melatonin (MEL)) phenolic conjugates in African violet petioles. Purple denotes 5HT conjugates (**a**), blue MEL conjugates (**b**–**d**). Bars represent mean, error bars span standard error margin. Where no error bars are displayed, the feature was present in only one replicate.

In addition to the presence of serotonin (5HT) itself in the control conditions, several oxidation/degradation products of 5HT and MEL were also detected including bufotenine, kynuramine and N1-acetyl-5-methoxykynuramine (AMK), along with 5-methyoxytryptamine (5-MT), an alternate biosynthetic pathway precursor for MEL (Figure 6). Both predicted amino acid and phenolic conjugates of MEL and 5HT were detected in tissues. 5HT-Trp (Figure 7a) was present only in the control, while 5HT-Arg (Figure 7b) and 5HT-Cys (Figure 7c) were both present only at 2 µM TDZ treatment. 5HT-Ser (Figure 7d) was absent in the control and increased with TDZ treatment. 5HT-Gln (Figure 7e) and 5HT-Asp (Figure 7f) were both present in all three treatments, however, 5HT-Asp was relatively stable while 5HT Gln was reduced by TDZ treatment but as much as half (2 µM TDZ). MEL-Asp was also present (Figure 7i) and along with MEL-Asn was detected only at 20 µM TDZ treatment while MEL-Lys (Figure 7g), the only other predicted MEL amino acid conjugated detected showed a linear increase with exposure to increasing levels of TDZ. Only one 5HT phenolic conjugate, N-coumaroylserotonin (Figure 8a) was detected in the control treatment, while three predicted MEL phenolic conjugates were detected: N-coumaroylmelatonin (Figure 8b), feruloylmelatonin (Figure 8c) and sinapoylmelatonin (Figure 8d). Ferulolymelatonin decreased with increase TDZ treatment while N-coumaroyl and sinapoyl melatonin were detected only at 2 µM or 20 µM TDZ, respectively.

## 4. Discussion

Though the broad applicability of TDZ in the induction of plant morphogenesis is now well established, the mechanisms which underpin the unique and dose-dependent morphogenetic outcomes of TDZ treatment are not well understood. This study has provided insights into both the metabolic fate of TDZ itself and TDZ-induced metabolism leading to organogenesis at low concentrations and somatic embryogenesis at high concentrations in the African violet regeneration system.

### 4.1. Metabolic Fate of TDZ in Plant Tissues

#### 4.1.1. Catabolism of TDZ

Our analysis did not detect features consistent with the TDZ monomer in African violet tissues and our synthetic biotransformation analysis indicated that the TDZ molecule is cleaved at the amide bridge releasing a thidiazol ring and aniline. Aniline is the product of metabolism of several herbicides and is incorporated into sugar and lignin conjugates [49]. Previous researchers have determined that derivatives of the 1,2,3-thidiazol-5yl ring are potent anti-senescence compounds [7]. Further catabolism of the thidiazol ring releases S and N into the tissue as organic compounds. Increased bioavailable S and N could drive the S-adenosylmethionine (SAM) cycle, which is responsible for production of nucleotides, cytokinins, polyamines, phospholipids, as well as increasing biosynthesis of other metabolites such as cysteine, serine, and glutathione. While the absolute amount of S and N released through metabolism of TDZ is quite low, it is possible that the bioavailability of the specific organic form accelerates targeted metabolism.

#### 4.1.2. Formation of Oligomers

The formation of TDZ oligomers in standard stock solutions has been previously proposed [2], and their presence in plant tissues is supported by the results of the current analysis, as masses consistent with the formation of dimer-conjugates and trimers are present. Structures of TDZ oligomers are not known but oligomerization may occur via a Diels–Alder reaction mechanism (Supplementary Figure S5). MM2 and MMFF94 energy minimization of the predicted structures in 3 dimensions provide insight into the loss of function observed with substitution of the phenol moiety [1]. Methods that detected the monomer to determine persistence in the environment [4] or in tissue culture media would not have detected oligomers of TDZ and therefore persistence in tissues and the environment may be underestimated. Further, oligomers of TDZ could provide an explanation for persistence of TDZ-induced responses following a short exposure to high concentrations [25,31,34].

#### 4.1.3. TDZ-Conjugates

Another explanation for the lack of intact TDZ in the tissues is the possibility that TDZ forms conjugates with other metabolites in plant cells. Ten compounds were discovered in TDZ-treated African violet explants that were entirely absent from control samples and could be conjugates of TDZ with other metabolites. Two of the potential TDZ forms contained the dimer of TDZ with O or Mg indicating the potential for TDZ to act as a ligand to metal ions or oxygen. Other potential TDZ-conjugates were polyphenols, peptides or fatty acids. We hypothesize that conjugates are storage forms of TDZ to deactivate or sequester the growth regulator.

### 4.2. TDZ-Induced Metabolism

#### 4.2.1. Primary Metabolism

Based on our pathway analysis indicating significant differences in metabolite profiles involved in core energy producing pathways such as the PPP and glycolysis in response to TDZ and the increased levels of 6C-phosphate and 7C sugars, we hypothesize that TDZ increases uptake and later catabolism of sugars from the medium. One of the first hypotheses for induction of regeneration was control of the uptake of nutrients from the culture media and maintenance of osmotic turgor in the isolated cells [50]. Sugar source-sink relationships are dependent on both passive and active transport across cell membranes and through plasmodesmata [51,52,53]. Our data suggest that TDZ may interact with one of the active sugar transporters to modulate uptake of 5C and 6C sugars from the culture media, with our results suggesting that it may favor uptake of 6C sugars. This is in agreement with previous studies, as for example, glucose was the only sugar source that when co-administered with TDZ led to high viability of poplar (*Populus tremula* x *P. alba*) protoplasts [54]. Co-application of TDZ and glucose has also been reported to induced somatic embryogenesis in cacao (*Theobrome cacao* L.) [55]. Increased overall carbohydrate accumulation has been reported in TDZ-induced bud break in apples (*Malus domestica* Borkh cv. Golden Delicious) [56] and has been associated with increased enzymatic activity of the TCA cycle, and PPP associated enzymes metabolizing 6C phosphates were found to be significantly enhanced during TDZ-induced budbreak of apple (*M. domestica*) [57].

Since the TDZ monomer is catabolized, it is possible that the TDZ molecule that interacts with the sugar transporter is the dimer, trimer or tetramer. Both carbon availability and macronutrient availability including nitrogen, phosphate and sulphate have been have been found to be an important factor in mediating not just growth, but also mediating regeneration and morphogenesis through accumulation of cytokinin precursors and modulation of expression of genes for cytokinin biosynthetic enzymes including isopentenyltransferase (IPT) [58,59,60].

#### 4.2.2. TDZ-Induced Terpene Metabolism

Our data indicate that TDZ has differential effects on terpene metabolism, generally promoting sesquiterpene and triterpene (farnesyl diphosphate, FPP) derived metabolites (e.g., triterpenes and triterpene saponins, brassinosteroids and phytosterols) and inhibiting diterpene (geranylgeranyl diphosphate, GGPP) derived metabolites (e.g., carotenoids, gibberellins, quinones). This is consistent with previous data that indicated an increase in fatty acids in peanuts treated with TDZ [61]. We hypothesize that TDZ inhibits biosynthesis of diterpene-derived metabolites and enhances synthesis of sesquiterpene and triterpene derivatives. Brassinosteroids and phytosterols which are both increased in response to TDZ treatment are FPP derivatives, while ABA and GA are both GGPP derived and were decreased, along with the carotenoids, which are precursors for ABA synthesis. Although both FPP and GGPP are derived from the hemiterpenes isopentyl diphosphate (IPP) and dimethylallyl diphosphate (DMAPP), the carbon inputs for the biosynthesis of these building block are sourced via two distinct pathways the deoxyxylulose (DXP)/methylerithrytol phosphate (MEP) pathway which is used in the case of GGPP and the mevalonate (MVA) pathway in the case of FPP. The DXP/MEP pathway utilizes glyceraldehyde-3-phosphate and pyruvate as the carbon sources while the mevalonate pathway utilizes two acetyl CoA unites [62,63]. Interestingly, the acetylCoA molecule contains a sulphur group and is derived from pyruvate from glycolysis, while G-3-P is a major intermediate of glycolysis is produced during the dark phase of photosynthesis and from fructose metabolism. It may therefore be increased availability of the carbon backbones which is partially controlling the carbon re-allocation to the terpene pathways with acetyl CoA being more available. Alternately TDZ may interact with important regulatory enzymes in these pathways such as 3-hydroxy-3-methylglutaryl CoA reductase (HMGR) the key regulatory step in the MVA pathway [64].

#### 4.2.3. TDZ-Induced Plant Growth Regulators

Differential effects on terpene metabolism could explain the observed changes in plant growth regulators derived from these pathways. TDZ-induced changes in GA and ABA metabolism could be responsible for some of the undesirable side effects of TDZ-induced organogenesis such as stunted shoots, malformed cotyledons, leaf swelling and other abnormalities [2,65]. Recently, brassinosteroids metabolism has also been implicated as TDZ upregulated several genes involved in brassinosteroid metabolism and signaling pathways including CYCD3-1 (CYCLIN D3-1; brassinosteroid and cytokinin signaling), BEE2 (BR enhanced expression 2; brassinosteroid signaling component) and DOGT1 (don-glucosyltransferase 1; brassinosteroid biosynthesis, glucosylation of brassinolide and castasterone) [66]. Surprisingly, our analysis did not detect any cytokinin or cytokinin related metabolites, which would have been expected based on their now well-established function in TDZ action. This may be simply due to limitations in our untargeted method, however, another possible explanation is that TDZ is inducing cytokinin-related pathways through interactions with cytokinin receptors as has been proposed by [1,67] vs. increasing levels of the cytokinins themselves in African violet, maintaining them at levels below our detection limits [1,67].

**Figure 9:**
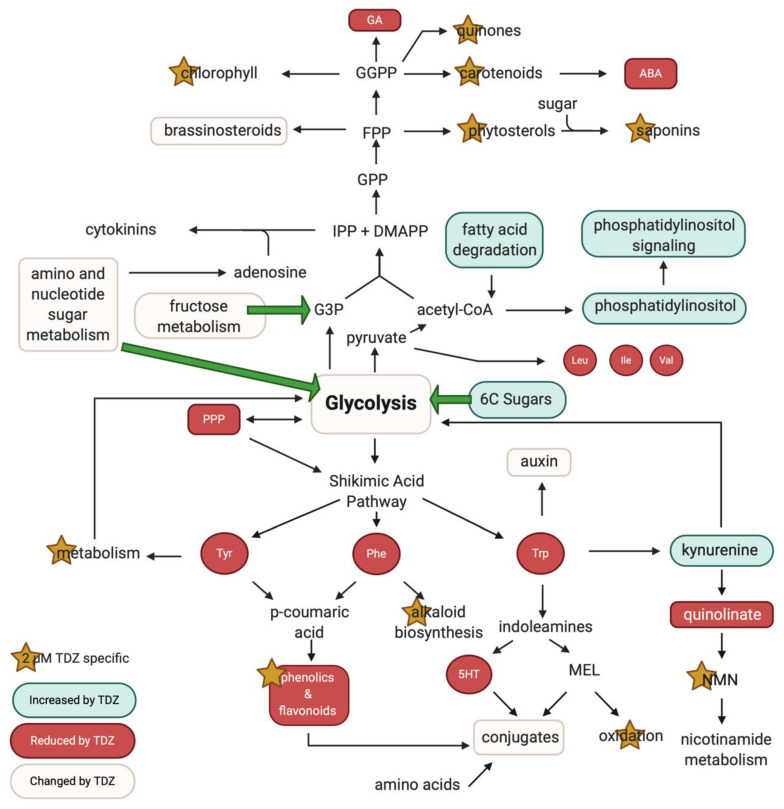
Thidiazuron (TDZ) mediation of glycolysis and shikimic acid derived metabolism. 5-HT, serotonin; ABA, abscisic acid; CS, coumaroylserotonin; DMAPP, dimethylallyl diphosphate; FPP, farnesyl diphosphate; GA, gibberellic acid; GGPP, geranyl geraniol diphosphate; IPP, isopentenyl diphosphate; MEL, melatonin; NMN, nicotinic acid mononucleotide; PPP, pentose phosphate pathway. Figure created in BioRender.

Pattern analysis of our untargeted data also detected changes in the shikimic acid pathway that generates the precursors for several classes of PGRs including auxin and the indoleamines (Figure 9). Tyr serves as a precursor for the catecholamines an emerging class of plant signaling molecules as well as the isoquinoline alkaloids and phenolics via conversion to p-coumaric acid via tyrosine ammonia lyase (TAL) [68]. Kynurenine is an important branch point in Trp catabolism that recycles carbon skeleton back into glycolysis suggesting that TDZ redirects carbon flow away from secondary metabolites and toward primary growth (Figure 9). Neither IAA nor its metabolite melatonin were detected in this analysis but this is not unexpected as both melatonin and auxin are expected to be unstable, degraded or oxidized in the extraction process [69]. However, metabolites and conjugates of indoleamine PGRs were found in the dataset, most notably the auxin precursor indol-3-acetamide. The observed decrease in indol-3-acetamide levels at 2 µM TDZ and increase in tissues treated with 20 µM TDZ is consistent with the previously hypothesized mechanisms of plant regeneration. At a high ratio of cytokinin to auxin, shoots are expected but when auxin and cytokinin levels are similar, somatic embryogenesis may be induced [2,24,26,70].

One of the most interesting observation in this study was the presence of a feature with the predicted formula C_35_H_52_O_4_ with the closest known match hyperforin but could also be a triterpene derivative isomer of hyperforin. C_35_H_52_O_4_ was not present in African violet explants in the absence of TDZ and increased linearly with increasing TDZ concentration (R^2^ = 0.9905) (Supplementary Figure S4). These data are consistent with the previous study that also putatively identified hyperforin and a cluster of related metabolites in a FT-MS dataset of *Scutellaria baicalensis* [41]. Conventional wisdom suggests that hyperforin is unique to species of the genus *Hypericum* and these results could indicate a more common occurrence during induction of plant regeneration. This is particularly interesting in light of reports that BAP-induced shoot induction is associated with accumulation of hyperforin in *Hypercium perforatum* [71]. Hyperforin accumulation may be a cytokinin-associated response and other phloroglucinols have been associated with increased shoot organogenesis and somatic embryogenesis [72].

### 4.3. TDZ-Induced Morphogenesis

**Figure 10:**
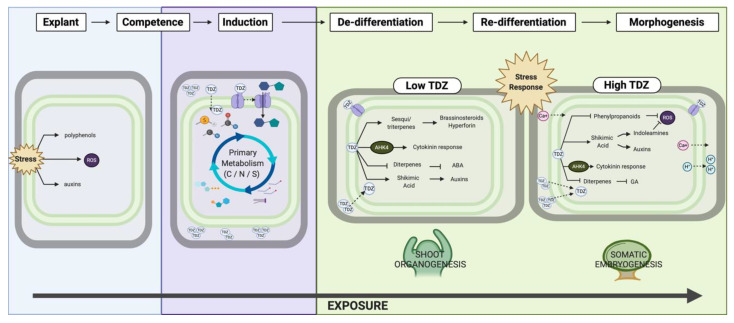
Proposed model for thidiazuron-induced morphogenesis in African violet. Figure created in BioRender.

The process of TDZ-induced morphogenesis can be broadly described as proceeding through (a) explanting, (b) induction, (c) growth and de-differentiation followed by either (d) shoot organogenesis or (e) somatic embryogenesis (Figure 6). The act of cleaving an explant from the maternal tissue creates a physical separation that disrupts the flow of metabolites between cells [73,74] including polar auxin transport, cytokinin flow and secondary messengers such as calcium signals. An inductive signal is then required to replace this continuum, often an exogenously applied cytokinin or auxin [70]. TDZ is a particularly effective PGR that can fulfill the role of the inductive signal of both auxins and cytokinins in many recalcitrant species [2]. Cells must be competent to accept the inductive signal and initiate a developmental pathway toward regeneration via organogenesis or embryogenesis (Figure 10) [51,73,74]. In diverse species such as geraniums, African violet and peanut (*Arachis hypogea*), TDZ induces somatic embryogenesis alone or in addition to shoot organogenesis [24,33,75,76,77]. Somatic embryogenesis is a typically auxin-associated growth outcome suggesting as has previously been hypothesized that TDZ action may proceed either via dual cytokinin and auxin-like activity [2,24,77]. The TDZ-induced morphogenesis may be dependent on secondary signals such as calcium [28,29] and/or physiological stress.

#### The Role of Stress

It has been hypothesized that the mode of action of TDZ may be through mediation of the physiological stress inherent in plant tissue culture [2,28,31]. While a certain level of stress is hypothesized to act as an inductive trigger, excess stress can be damaging through increased reactive oxygen species and phenolic accumulation. TDZ has been found to enhance antioxidant capacity in diverse species as well as mediating phenolic accumulation [18] and, in this study, low levels of TDZ increased glutathione while higher levels decreased 5HT and MEL through increased conjugation (Figure 9). MEL and 5HT have been associated with a diversity of plant morphogenetic responses [78,79,80], to contribute to plant antioxidant capacity and to interact with plant phenolic pathways [81], but have not previously been described in African violets. It is possible that induction of indoleamines by high levels of TDZ treatment associated with somatic embryogenesis may reflect the multi-dimensional functions of TDZ supporting the stress protection and growth of tissues while simultaneously supporting a physiological environment conducive to embryo development which explains its auxin-like activity [29,77]. While the accumulation and subsequent slow release of conjugated TDZ could be is responsible for the embryogenic growth in a mechanism analogous to the induction of somatic embryogenesis by auxins [24,77], the mode of action of auxin-induced embryogenesis itself is still poorly understood. An alternate hypothesis to conjugation or sequestration has been the presence of a modifying or co-protective compound which otherwise may not be available to the tissues. This protective auxin speculated by Street (1979) may be the indoleamines Mel and/or 5HT which contribute to protection of tissues in addition to auxin-cytokinin both for induction of somatic embryogenesis [82].

## 5. Conclusions

Despite more than 40 years of use, widespread application in the environment and hundreds of publications demonstrating TDZ-induced plant morphogenesis, the exact mechanism of action remains unknown. Studies that searched for specific binding to an individual receptor or enzyme have failed to provide satisfactory explanations for the diversity of metabolic responses. Metabolomics and hormonomics approaches have the potential to unravel the mysteries of TDZ-induced metabolism thereby providing new insights into the mechanisms of plant growth and regeneration. Here we have utilized this approach to develop six hypotheses for the mechanism of TDZs action in modulation organogenesis and somatic embryogenesis in the petioles of African violet:

1. TDZ is metabolized by plant cells to release bioavailable sulfur and nitrogen.
2. TDZ forms oligomers in solutions and plant tissues.
3. TDZ forms conjugates as inactive or storage forms of TDZ.
4. TDZ increases uptake and catabolism of 5C and 6C sugars from the culture medium.
5. TDZ inhibits biosynthesis of diterpene-derived metabolites and enhances synthesis of sesquiterpene and triterpene derivatives.
6. TDZ mediates the rate of flux of metabolites through the shikimic acid pathway producing plant growth regulators and secondary metabolites.

Investigation of these hypotheses will lead to new understandings of the mode of action of TDZ and its regulatory role in plant morphogenesis.

## Figures and Tables

**Table 1:** Features identified as significant ^a^ modulated in response to thidiazuron treatment as compared to control according to partial least squares-discriminant analysis (PLS-DA) VIP Scores results. Metabolite mass is the predicted monoisotopic mass of the compound, M, monoisotopic mass is the known monoisotopic mass (as predicted by ChemSpider). Data is presented as mean ion intensity. Shading darkens with increasing levels.

Metabolite Mass	Putative ID *	Class	Associated KEGG Metabolic Pathway(s)	M	0	2	20
254.05791	(Z)-4′,6-Dihydroxyaurone	Aurone flavonoid	Flavonoid biosynthesis Isoflavonoid biosynthesis	254.0579	62336170	35136500	18638300
	Chrysophanol	Anthraquinone					
	Chrysin	Flavone					
	7,4′-Dihydroxyflavone	Flavone					
	Anhydroglycinol	Pterocarpan					
	Daidzein	Isoflavone					
	formononetin methylated	Isoflavone					
	5,7-Dihydroxyisoflavone	Isoflavone					
254.058126	(Z)-4′,6-Dihydroxyaurone	Aurone flavonoid	Flavonoid biosynthesis Isoflavonoid biosynthesis	254.0579	62336170	35136500	18638300
	Chrysophanol	Anthraquinone					
	Chrysin	Flavone					
	7,4′-Dihydroxyflavone	Flavone					
	Anhydroglycinol	Pterocarpan					
	Daidzein	Isoflavone					
	formononetin methylated	Isoflavone					
	5,7-Dihydroxyisoflavone	Isoflavone					
297.266779	Palmitoleoyl ethanolamide	N-acylethanolamines	Glycerophosphate metabolism	297.2668	29559440	19908290	10659625
202.045236	Ethyl aconitate	Tricarboxylic acid derivative	Citrate Cycle	202.0477	83643600	54038350	64287550
364.09741	Gerberinol	4-Hydroxycoumarin	Phenylpropanoid biosynthesis/Biosynthesis of phenylpropanoids Phenylpropanoid biosynthesis	364.0947	64478035	54169390	78307450
230.076354	Aspartyl-Proline	Dipeptide	Arginine and proline metabolism Glutathione metabolism	230.0903	17390700	22240300	30965200
	(2S,3′S)-alpha-Amino-2-carboxy-5-oxo-1-pyrrolidinebutanoic acid	Proline derivative					
	Prolyl-Aspartate	Dipeptide					
	1-(gamma-Glutamylamino)cyclopropanecarboxylic acid	Dipeptide					
	Aspartyl-L-proline	Dipeptide					
230.076536	Aspartyl-Proline	Dipeptide	Arginine and proline metabolism Glutathione metabolism	230.0903	17390700	22240300	30965200
	(2S,3′S)-alpha-Amino-2-carboxy-5-oxo-1-pyrrolidinebutanoic acid	Proline derivative					
	Prolyl-Aspartate	Dipeptide					
	1-(gamma-Glutamylamino)cyclopropanecarboxylic acid	Dipeptide					
	Aspartyl-L-proline	Dipeptide					
278.094295	Methionyl-Glutamate	Dipeptide	Glutathione metabolism	278.0936	24758300	26486100	13594000
	Glutamylmethionine	Dipeptide					
	N-gamma-L-Glutamyl-L-methionine	Dipeptide					
278.094511	Methionyl-Glutamate	Dipeptide	Glutathione metabolism	278.0936	24758300	26486100	13594000
	Glutamylmethionine	Dipeptide					
	N-gamma-L-Glutamyl-L-methionine	Dipeptide					
278.09498	Methionyl-Glutamate	Dipeptide	Glutathione metabolism	278.0936	24758300	25678000	13594000
	Glutamylmethionine	Dipeptide					
	N-gamma-L-Glutamyl-L-methionine	Dipeptide					
612.475375	DG	Diacylglycerol	>10	612.4754	26360600	22255850	17457400
364.097879	Gerberinol	4-Hydroxycoumarin	Phenylpropanoid biosynthesis Biosynthesis of phenylpropanoids Phenylpropanoid biosynthesis	364.0947	49914200	42166150	58372250
364.098061	Gerberinol	4-Hydroxycoumarin	Phenylpropanoid biosynthesis/Biosynthesis of phenylpropanoids Phenylpropanoid biosynthesis	364.0947	49824750	42156490	57558100
364.098277	Gerberinol	4-Hydroxycoumarin	Phenylpropanoid biosynthesis/Biosynthesis of phenylpropanoids Phenylpropanoid biosynthesis	364.0947	49824750	42220140	57474850

* Closest match in FooDB, excluding those compounds which have not previously been reported to be present in plants; for compounds with multiple possible functional group positions only one has been included. ^a^ Top 30 features included, excluding features which have no reasonable match in FooDB.

**Table 2:** Metabolites that were not detected in controls but present in thidiazuron (2 or 20 µM) treated tissues. Metabolite mass is the predicted monoisotopic mass of the compound, monoisotopic mass is the known monoisotopic mass (as predicted by ChemSpider). Data is presented as mean ion intensity. Regression coefficient (R^2^) was determined for the linear relationship of 20 µM > 2 µM > 0. Shading darkens with increasing levels.

Metabolite Mass	Putative ID *	Class	Associated KEGG Metabolic Pathway(s)	Monoisotopic Mass	0	2	20	R^2^ (Linear) ^a^
162.14085	Pregeijerene	Unsaturated Hydrocarbon		162.1408506	0	846985	862490	0.347
	1,3-Diisopropylbenzene	Aromatic hydrocarbon		162.1408506				
192.027005	dehydroascorbate (bicyclic form)	Ascorbate	Pentose and glucoronate interconversions Ascorbate and aldarate metabolism	192.0270026	0	524910	502955	0.298
	Isocitric acid	Ascorbate		192.0270026				
	Citric acid	Ascorbate		192.0270026				
	2,3-Diketo-L-gulonate	Ascorbate degradation		192.0270026				
	D-Glucaro-1,4-lactone	Monosaccharide		192.0270026				
196.109945	Loliolide	carotenoid metabolite; benzofuran	Furfural degradation	196.1099444	0	1036230	902080	0.226
	3-Methylidene-4-oxopentyl angelate	Fatty acid ester		196.1099444				
	Isobutyl 2-furanpropionate	Fatty acid ester		196.1099444				
	Hexyl 2-furoate	Fuoric acid ester		196.1099444				
270.052825	4,6-dihydroxy-2-[(4-hydroxyphenyl)methylidene]-2,3-dihydro-1-benzofuran-3-one	Aurone flavonoid	Flavonoid biosynthesis Isoflavonoid biosynthesis Biosynthesis of phenylpropanoids	270.0528234	0	1392765	1071285	0.146
	Rheinanthrone	Anthracenecarboxylic acid		270.0528234				
	Aloeemodin	Anthraquinone		270.0528234				
	Rhababerone	Anthraquinone		270.0528234				
	1,2,8-Trihydroxy-3-methylanthraquinone	Anthraquinone		270.0528234				
	Emodin	Anthraquinone		270.0528234				
	Apigenin	Flavone		270.0528234				
	Norizalpinin	Hydroxyflavonol		270.0528234				
	3,4′,7-Trihydroxyflavone	Hydroxyflavonol		270.0528234				
	6-Hydroxydaidzein	Isoflavone		270.0528234				
	Genistein	Isoflavone		270.0528234				
330.122909	Tyramine-betaxanthin	Betalain	Betalain biosynthesis	330.1216	0	924825	892190	0.303
352.068037	a-L-threo-4-Hex-4-enopyranuronosyl-D-galacturonic acid	Glucoronic acid derivative	Pentose and glucoronate interconversions	352.064176	0	1220300	1574900	0.545
406.107975	(R)-Apiumetin glucoside	Coumarin glycoside	Tyrosine metabolism Benzoate degradation Flavonoid biosynthesis	406.1263823	0	578125	1001885	0.752
	Edulisin VI	Furanocoumarin		406.1263823				
	Benzyl 2,6-dihydroxybenzoate 2-glucoside	Phenolic glycoside		406.1263823				
	omega-Salicoyisalicin	Phenolic glycoside		406.1263823				
	Flacourtin	Phenolic glycoside		406.1263823				
	Dihydroresveratrol 3-glucuronide	Stilbene glycoside		406.1263823				
	Piceatannol 4′-glucoside	Stilbene glycoside		406.1263823				
	(E)-Oxyresveratrol 3′-O-b-D-glucoside	Stilbene glycoside		406.1263823				
	Astringin	Stilbene glycoside		406.1263823				
500.31379	Theaflavin 3,3′-digallate	Polyphenol	Flavonoid biosynthesis Brassinosteroid biosynthesis Sesquiterpenoid and triterpenoid biosynthesis	500.3148863	0	1350235	2631535	0.813
	Physalolactone B	Sterol		500.3148863				
	medicagenate	Triterpene		500.3148863				
	Ganolucidic acid A	Triterpene		500.3148863				
	Ganoderic acid beta	Triterpene derivative		500.3148863				
520.101648	Melitric acid B	Polyphenol	Phenylpropanoid biosynthesis	520.1005615	0	442050	511095	0.454
536.38656	Hyperforin	Polycyclic Polyprenylated Acylphloroglucinol	Sesquiterpenoid and triterpenoid biosynthesis Aminobenzoate degradation	536.3865392	0	1302600	6563250	0.990
568.119495	Neobignonoside	Flavonoid glycoside	Flavonoid biosynthesis Polyphenol biosynthesis Biosynthesis of secondary metabolites	568.1216909	0	403535	591625	0.643
	Chrysophanol 8-(6-galloylglucoside)	Anthraquinone		568.1216909				
570.155971	Apiumoside	Psoralen	Biosynthesis of phenylpropanoids	570.1737264	0	1204850	1155410	0.299
616.503989	DG	Diacylglycerol	>10	616.5066753	0	461850	446055	0.304
636.473874	DG	Diacylglycerol	>10	636.4753752	0	318400	380860	0.483
684.234252	Maltulose	Glycerolipid	Glycerolipid metabolism	684.2324231	0	600440	1097880	0.781
	Galabiose	Glycerolipid		684.2324231				
694.377879	Capsoside A	Glycosyldiacylglycerol	Glycerolipid metabolism	694.3775712	0	551420	567555	0.356
778.41126	Periandrin V	Triterpene glycoside	Sesquiterpenoid and triterpenoid metabolism	778.4139567	0	835800	810640	0.308
810.437975	9-Hentriacontanone	Ketone	Sesquiterpenoid and triterpenoid metabolism	810.4401714	0	3421550	4660950	0.588
	Phytolaccoside D2	Triterpene		810.4401714				
	Phytolaccoside D	Triterpene		810.4401714				
	Elatoside H	Triterpene saponin		810.4401714				
	Cynarasaponin E	Triterpene saponin		810.4401714				
	Lucyoside J	Triterpene saponin		810.4401714				
	Azukisaponin III	Triterpene saponin		810.4401714				
	Spinasaponin B	Triterpene saponin		810.4401714				
880.752675	TG	Triacylglycerol	Glycerolipid metabolism	880.7519909	0	2626300	2252500	0.215

* Closest match in FooDB, excluding those compounds which have not previously been reported to be present in plants; for compounds with multiple possible functional group positions only one has been included. ^a^ R^2^ highlighted where > 0.75.

**Table 3:** Metabolites present in thidiazuron (TDZ) treated tissues and absent in control predicted to have been presented as conjugates of thidiazuron (TDZ). Metabolite mass is the predicted monoisotopic mass of the compound, monoisotopic mass is the known monoisotopic mass (as predicted by ChemSpider). Data is presented as mean ion intensity. M-TDZ is the predicted monoisotopic mass of the compound less the monoisotopic mass of one TDZ molecule (220.041882); M-2TDZ is the predicted monoisotopic mass of the compound less the monoisotopic mass of two TDZ molecules. Regression coefficient (R^2^) was determined for the linear relationship of 20 µM > 2 µM > 0. Shading darkens with increasing levels.

Metabolite Mass	M-TDZ	M-2TDZ	Putative ID *	Class	Monoisotopic Mass	Formula	0	2	20	R^2^ (Linear)
432.072153	212.030271		4-Nitroso-2,6-dinitrotoluene	Dinitrotoluene	211.0229	C7H5N3O5	0	409980	585255	0.624
			2-Methyl-1,5-dinitro-3-nitrosobenzene	Dinitrotoluene	211.0229	C7H5N3O5				
		7.003782	No match							
456.0691	236.027218		No match				0	480545	712690	0.652
		16.993165	Oxygen	Element	15.999	O				
465.049091	245.007209		Coumaric acid sulfate	Phenylpropanoid	244.0042	C9H8O6S	0	813650	1281750	0.693
		25.973156	Magnesium	element	24.305	Mg				
676.200542	456.15866		5,10-Methenyltetrahydrofolate	Tetrahydrofolic acid	455.1553	C20H21N7O6	0	6572500	5052450	0.146
		237.124607	Phenylalanylalanine	Dipeptide	236.1161	C12H16N2O3				
706.21458	486.172698				485.594	C29H27NO4S **	0	1335030	2554800	0.804
		267.138645	3-Phenylpropyl cinnamate	Cinnamic acid ester	266.1307	C18H18O2				
718.210422	498.16854				498.55474	C23H26N6O5S **	0	1729150	1555300	0.250
		279.134487	Tyrosyl proline	Dipeptide	278.1267	C14H18N2O4				
			L-Phenylalanylyl-L-hydroxyproline	Dipeptide	278.1267	C14H18N2O4				
726.359148	506.317266		LysoPE	Llysophospholipid	505.3168	C25H48NO7P	0	1226900	864805	0.101
		287.283213	Hexanal dihexyl acetal	Acetal	286.2872	C18H38O2				
760.488734	540.446852				539.794	C29H57N5O4	0	473410	578200	0.500
		321.412799								
826.411427	606.369545				605.8403	C32H47N9OS **	0	1501580	1451550	0.305
		387.335492	1-Phenyl-1,3-eicosandeione	Alkyl phenylketone	386.3185	C26H42O2				
961.423907	741.382025					C38H56N6O5S2 **	0	1241850	1256550	0.342
		522.347972	LysoPC	Lysophospholipid	521.3481	C26H52NO7P				
			Lysolecithin	Phosphatidylcholine	521.3481	C26H52NO7P				

* Closest match in FooDB, excluding those compounds which have not previously been reported to be present in plants; for compounds with multiple possible functional group positions only one has been included. ** Putative chemical formula based on a search of the Chem Spider database, where no likely FooDB matches are present.

**Table 4:** Results of pathway analysis results for African violet petiole cultures treatment with 0 vs. 2 µM thidiazuron (low TDZ) and 0 vs. 20 µM thidiazuron (high TDZ). To provide an overall picture of metabolic pathways modulated by treatment results of pairwise t-test and volcano analysis or Pattern Hunter analysis was used as the input for pathway analysis by mummichog and GSEA (t-test only) in peaks to pathways.

Test	Comparison/Pattern	Pathways Significantly Modulated	Combined *p*-Values
***t*-test**	0 vs. 2 µM TDZ	Steroid biosynthesis Folate biosynthesis Histidine metabolism	
	0 vs. 20 µM TDZ	Steroid biosynthesis Sesquiterpene and triterpene biosynthesis	
**Volcano Analysis**	0 vs. 2 µM TDZ	Purine biosynthesis Starch and sucrose metabolism Steroid biosynthesis	
	0 vs. 20 µM TDZ	Glycolysis/gluconeogenesis Pentose phosphate pathway Fructose and mannose metabolism Galactose metabolism Fatty acid degradation	
**Pattern Hunter**	Linear Increase 0 < 2 < 20 µM TDZ	N-glycan biosynthesis	0.01
		Fructose and mannose metabolism	0.02
		Phosphatidylinositol signaling	0.03
		Amino sugar and nucleotide sugar metabolism	0.04
		Inositol phosphate metabolism	0.04
		Arachidonic acid metabolism	0.04
		Glycolysis/gluconeogenesis	0.05
		Galactose metabolism	0.05
	Linear Decrease 0 > 2 > 20 µM TDZ	Porphyrin and chlorophyll metabolism	0
		Phosphatidylinositol signaling	0.01
		Starch and sucrose metabolism	0.01
		Galactose metabolism	0.01
		Inositol phosphate metabolism	0.01
		Glycolysis/gluconeogenesis	0.02
		Pentose phosphate pathway	0.02
		Valine, leucine, isoleucine Biosynthesis	0.02
		Fructose and mannose Metabolism	0.02
		Ascorbate and aldarate Metabolism	0.03
		Caffeine metabolism	0.04
		Amino sugar and nucleotide Sugar metabolism	0.04
		Phenylalanine, tyrosine and tryptophan biosynthesis	0.05
		Flavonoid biosynthesis	0.05
		Pentose and glucoronate interconversions	0.05
	Peak at 2 µM TDZ 2 > 20 > 0	Porphyrin and chlorophyll metabolism	0
		Tyrosine metabolism	0.02
		Tropane, piperidine and pyridine alkaloid biosynthesis	0.03
		Sesquiterpenoid and triterpenoid biosynthesis	0.05
	Dip at 2 µM TDZ 2 < 20 < 0	Carotenoid biosynthesis	0.05
		Galactose metabolism	0.05
		Flavonoid biosynthesis	0.05

**Table 5:** Results of significance analysis of microarrays (SAM) and empirical Bayes analysis of microarrays (EBAM) analysis results for African violet cultures treatment with 0 vs. 2 µM thidiazuron (low TDZ), 0 vs. 20 µM thidiazuron (high TDZ) yielded no significant results and is not displays. Results of pairwise results were searched in FooDB for matches.

Test	Putative FooDB ID	Metabolite Class	Associated KEGG Pathway(s)
**SAM**	Triterpene saponin Lucyoside M Spinasaponin A Cynarasaponin C Calendulaglucoside E	Triterpene saponin	Sesquiterpenoid and triterpenoid biosynthesis
**EBAM**	Gerberinol	4-Hydroxycoumarin	Phenylpropanoid biosynthesis/Biosynthesis of phenylpropanoids Phenylpropanoid biosynthesis
	Xanthotoxol glucoside	Coumarin glucoside	Biosynthesis of secondary metabolites
	1-Methyl-3-(2-thiazolyl)-1H-indole	Camalexin derivative	MAPK Signaling pathway-plant
	Triglyceride	Lipid	Triacylglycerol biosynthesis
	a-L-threo-4-Hex-4-enopyranuronosyl-D-galacturonic acid	Glucuronic acid derivative	Pentose and glucoronate interconversions Ascorbate and aldarate metabolism Amino sugar and nucleaotide sugar metabolism Inositol phosphate metabolism
	4-Methylumbelliferone glucuronide	Glucoronic acid derivative/hydroxycoumarin	
	Chlorogenoquinone	Quinic acid derivative	Phenylalanine, tyrosine and tryptophan biosynthesis

**Table 6:** Effect of thidiazuron treatment on sugar uptake and metabolism in petioles of African violet. Data are displayed as percent of control total ion count, where control is considered 100%. Coloring indicates increasing (green) or decreasing metabolites (blue) with highest decreases or increases in darker tones.

Putative ID	Metabolite Mass	Monoisotopic Mass	2 µM TDZ (% of Control)	20 µM TDZ (% of Control)
Salicin phosphate	366.0716	366.073955	150.16	200.33
Disaccharide	342.1162	342.115732	85.58	116.90
Arbutin	272.0896	272.087101	76.67	277.72
Arbutin-phosphate	352.0559	352.068037	74.68	93.34
Salicin	286.1053	286.09	42.09	51.85
Glucose/fructose	180.0634	180.06339	88.79	92.48
2-Dehydro-d-gluconate	194.0427	194.05791	69.25	74.66
6C Disaccharide-P	422.0825	422.082547	60.09	92.73
5C Monosaccharide-P	230.0192	230.019157	72.19	50.21
6C Monosaccharide-P	260.0297	260.029722	605.62	55.80
7C Monosaccharide-P	290.0403	290.049016	113.01	49.21
deoxy-ribose-P	214.0242	214.008851	101.84	91.18

## Supplementary Materials

The following are available online at https://www.mdpi.com/2218-273X/10/9/1253/s1, Figure S1: Thidiazuron (TDZ) induced morphogenesis in African violet (a) and (b) shown organogenesis induced by 2 µM TDZ, (c) somatic embryogenesis induced by 10 µM TDZ, Figure S2: (a–c) Principle component analysis (PCA) biplots for principle components (PCs) 1 through 3. (d–f) Partial least squares-discriminant analysis (PLS-DA) biplots for components 1–3 for African violet petioles treated with 0, 2 or 20 µM thidiazuron, Figure S3: Cross validation plots for PLS-DA analysis, Figure S4: Diterpenes and sesquiterpenes accumulate in TDZ-exposed tissues. Gray line is putatively identified as hyperforin (structure inset), Figure S5: Potential Diels-Alder mechanisms of formation of dimers and trimers of thidiazuron (TDZ) in plant cells. (A) dimer; (B) trimer; (C) tetramer, Table S1: Standard Operating Protocol methods of extraction and ionization of metabolites as described by [40], Table S2: Summary of query masses for hormonomics analysis. 5HT, serotonin; ABA, abscisic acid; IAA, indole-3-acetic acid; JA, jasmonic acid; MEL, melatonin; Trp, tryptophan, Table S3: Summary of metabolomic database.

## References

[1] Mok M.C., Mok D.W.S., Armstrong D.J., Shudo K., Isogai Y., Okamoto T. (1982). Cytokinin activity of N-phenyl-N′-1, 2,3-thiadiazol-5-ylurea (thidiazuron). Phytochemistry.

[2] Murthy B.N.S., Murch S.J., Saxena P.K. (1998). Thidiazuron: A potent regulator of in vitro plant morphogenesis. Vitro Cell. Dev. Biol. Plant.

[3] Xu J., Chen L., Sun H., Wusiman N., Sun W., Li B., Gao Y., Kong J., Zhang D., Zhang X. (2019). Crosstalk between cytokinin and ethylene signaling pathways regulates leaf abscission in cotton in response to chemical defoliants. J. Exp. Bot..

[4] Xin F., Zhao J., Zhou Y., Wang G., Han X., Fu W., Deng J., Lan Y. (2018). Effects of Dosage and Spraying Volume on Cotton Defoliants Efficacy: A Case Study Based on Application of Unmanned Aerial Vehicles. Agronomy.

[5] Xu J.H., Li C.P., Liu Z.S., Liu S.J., Zhang D.W., Ning X.M., Xie D.J. (2011). Breeding and planting techniques of the high-quality and high-yield new variety Xinluzao 50. China Cotton.

[6] Gormus O., Kurt F., Sabagh A.E. (2017). Impact of defoliation timings and leaf pubescence on yield and fiber quality of cotton. J. Agric. Sci. Technol..

[7] Nisler J., Zatloukal M., Sobotka R., Pilný J., Zdvihalová B., Novák O., Strnad M., Spíchal L. (2018). New Urea derivatives are effective anti-senescence Compounds acting most likely via a cytokinin-Independent mechanism. Front. Plant Sci..

[8] Toscano S., Trivellini A., Ferrante A., Romano D. (2018). Physiological mechanisms for delaying the leaf yellowing of potted geranium plants. Sci. Hortic..

[9] Famiani F., Proietti P., Pilli M., Battistelli A., Moscatello S. (2007). Effects of application of thidiazuron (TDZ), gibberellic acid (GA 3), and 2,4-dichlorophenoxyacetic acid (2,4-D) on fruit size and quality of Actinidia deliciosa ‘Hayward’. N. Z. J. Crop Hortic. Sci..

[10] Stern R., Shargal A., Flaishman M. (2003). Thidiazuron increases fruit size of ‘Spadona’ and ‘Coscia’ pear (Pyrus communis L.). J. Hortic. Sci. Biotechnol..

[11] Reynolds A., Wardle D., Zurowski C., Looney N. (1992). Phenylureas CPPU and Thidiazuron Affect YieldComponents, Fruit Composition, and Storage Potential of Four Seedless Grape Selections. J. Am. Soc. Hortic. Sci..

[12] Greene D.W. (1995). Thidiazuron Effects on Fruit Set, Fruit Quality, and Return Bloom of Apples. Hortscience.

[13] Do Amarante C.V.T., Megguer C.A., Blum L.E.B. (2003). Effect of preharvest spraying with thidiazuron on fruit quality and maturity of apples. Rev. Bras. Frutic..

[14] Do Amarante C.V.T., Ernani P.R., Blum L.E.B., Megguer C.A. (2002). Thidiazuron effects on shoot growth, return bloom, fruit set and nutrition of apples. Pesq. Agropec. Bras..

[15] Pasa M.S., Silva C.P.D., Carra B., Brighenti A.F., Souza A.L.K.D., Petri J.L. (2017). Thidiazuron (TDZ) increases fruit set and yield of ‘Hosui’ and ‘Packham’s Triumph’ pear trees. An. Acad. Bras. Ciênc..

[16] Yang Y.-Z., Lin D.-C., Guo Z.-Y. (1992). Promotion of fruit development in cucumber (Cucumis sativus) by thidiazuron. Sci. Hortic..

[17] Lu C.-Y. (1993). The use of thidiazuron in tissue culture. Vitro Cell. Dev. Biol. Plant.

[18] Guo B., Abbasi B.H., Zeb A., Xu L.L., Wei Y.H. (2011). Thidiazuron: A multi-dimensional plant growth regulator. Afr. J. Biotechnol..

[19] Debnath S.C., Ahmad N., Faisal M. (2018). Hidiazuron in Micropropagation of Small Fruits. Thidiazuron: From Urea Derivative to Plant Growth Regulator.

[20] Huetteman C.A., Preece J.E. (1993). Thidiazuron: A potent cytokinin for woody plant tissue culture. Plant Cell Tissue Organ Cult..

[21] Hare P.D., Staden J.V. (1994). Inhibitory Effect of Thidiazuron on the Activity of Cytokinin Oxidase Isolated from Soybean Callus. Plant Cell Physiol..

[22] De Ferreira W.M., Kerbauy G.B., Kraus J.E., Pescador R., Suzuki R.M. (2006). Thidiazuron influences the endogenous levels of cytokinins and IAA during the flowering of isolated shoots of Dendrobium. J. Plant Physiol..

[23] Hothorn M., Dabi T., Chory J. (2011). Structural basis for cytokinin recognition by Arabidopsis thaliana histidine kinase 4. Nat. Chem. Biol..

[24] Visser C., Qureshi J.A., Gill R., Saxena P.K. (1992). Morphoregulatory role of thidiazuron substitution of auxin and cytokinin requirement for the induction of somatic embryogenesis in geranium hypocotyl cultures. Plant Physiol..

[25] Hutchinson M.J., Saxena P.K. (1996). Acetylsalicylic acid enhances and synchronizes thidiazuron-induced somatic embryogenesis in geranium (Pelargonium x hortorum Bailey) tissue cultures. Plant Cell Rep..

[26] Naseem A., Mohammad F. (2018). Thidiazuron: From Urea Derivative to Plant Growth Regulator.

[27] Murch S.J., Saxena P.K. (2001). Molecular fate of thidiazuron and its effects on auxin transport in hypocotyls tissues of *Pelargonium × hortorum* Bailey. Plant Growth Regul..

[28] Jones M.P.A., Yi Z., Murch S.J., Saxena P.K. (2007). Thidiazuron-induced regeneration of Echinacea purpurea L.: Micropropagation in solid and liquid culture systems. Plant Cell Rep..

[29] Murch S.J., Victor J.M.R., Saxena P.K. (2003). Auxin, Calcium and Sodium in Somatic Embryogenesis of African Violet. Acta Hortic..

[30] Hutchinson M.J., Senaratna T., Sahi S.V., Saxena P.K. (2000). Light Mediates Endogenous Plant Growth Substances in Thidiazuron-induced Somatic Embryogenesis in Geranium Hypocotyl Cultures. J. Plant Biochem. Biotechnol..

[31] Murch S.J., Krishnaraj S., Saxena P.K. (1997). Thidiazuron-induced morphogenesis of Regal geranium (Pelargonium domesticum): A potential stress response. Physiol. Plant.

[32] Mithila J., Hall J., Victor J.M.R., Saxena P. (2003). Thidiazuron induces shoot organogenesis at low concentrations and somatic embryogenesis at high concentrations on leaf and petiole explants of African violet (Saintpaulia ionantha Wendl.). Plant Cell Rep..

[33] Padmanabhan P., Murch S.J., Sullivan J.A., Saxena P.K. (2015). Micropropagation of Primulina dryas (Dunn) Mich. Möller & A. Webber: High frequency regeneration from leaf explants. Sci. Hortic..

[34] Padmanabhan P., Sullivan J.A., Murch S.J., Saxena P. (2014). Development of an efficient protocol for high frequency in vitro regeneration of a horticultural plant Primulina tamiana (B.L. Burtt) Mich. Möller & A. Webber. Can. J. Plant Sci..

[35] Fiehn O., Kopka J., Dörmann P., Altmann T., Trethewey R.N., Willmitzer L. (2000). Metabolite profiling for plant functional genomics. Nat. Biotechnol..

[36] Turi C.E., Finley J., Shipley P.R., Murch S.J., Brown P.N. (2015). Metabolomics for Phytochemical Discovery: Development of Statistical Approaches Using a Cranberry Model System. J. Nat. Prod..

[37] Murthy B.N., Singh R.P., Saxena P.K. (1996). Induction of high-frequency somatic embryogenesis in geranium (Pelargonium x hortorum Bailey cv Ringo Rose) cotyledonary cultures. Plant Cell Rep..

[38] Shukla M., Sullivan J.A., Jain S.M., Murch S.J., Saxena P.K. (2013). Micropropagation of African violet (Saintpaulia ionantha Wendl.). Methods Mol. Biol..

[39] Aharoni A., de Vos C.H.R., Verhoeven H.A., Maliepaard C.A., Kruppa G., Bino R., Goodenowe D.B. (2002). Nontargeted Metabolome Analysis by Use of Fourier Transform Ion Cyclotron Mass Spectrometry. OMICS J. Integr. Biol..

[40] Hirai M.Y., Yano M., Goodenowe D.B., Kanaya S., Kimura T., Awazuhara M., Arita M., Fujiwara T., Saito K. (2004). From the Cover: Integration of transcriptomics and metabolomics for understanding of global responses to nutritional stresses in Arabidopsis thaliana. Proc. Natl. Acad. Sci. USA.

[41] Murch S.J., Rupasinghe H.P.V., Goodenowe D., Saxena P.K. (2004). A metabolomic analysis of medicinal diversity in Huang-qin (Scutellaria baicalensis Georgi) genotypes: Discovery of novel compounds. Plant Cell Rep..

[42] Murch S.J., Saxena P.K. (2006). A melatonin-rich germplasm line of St John’s wort (Hypericum perforatum L.). J. Pineal Res..

[43] Xia J., Psychogios N., Young N., Wishart D.S. (2009). MetaboAnalyst: A web server for metabolomic data analysis and interpretation. Nucleic Acids Res..

[44] Chong J., Soufan O., Li C., Caraus I., Li S., Bourque G., Wishart D.S., Xia J. (2018). MetaboAnalyst 4.0: Towards more transparent and integrative metabolomics analysis. Nucleic Acids Res..

[45] Xia J., Wishart D.S. (2011). Web-based inference of biological patterns, functions and pathways from metabolomic data using MetaboAnalyst. Nat. Protoc..

[46] Erland L., Turi C.E., Saxena P.K., Murch S.J. (2020). Metabolomics and hormonomics to crack the code of filbert growth. Metabolomics.

[47] Brown P., Turi C., Shipley P., Murch S.J. (2012). Comparisons of Large (Vaccinium macrocarponAit.) and Small (Vaccinium oxycoccos L., Vaccinium vitis-idaea L.) Cranberry in British Columbia by Phytochemical Determination, Antioxidant Potential, and Metabolomic Profiling with Chemometric Analysis. Planta Med..

[48] Turi C.E., Murch S.J. (2013). Targeted and untargeted phytochemistry of Ligusticum canbyi: Indoleamines, phthalides, antioxidant potential, and use of metabolomics as a hypothesis-generating technique for compound discovery. Planta Med..

[49] Lieb H.E., Still C.C. (2020). Herbicide Metabolism in Plants: Specificity of peroxidases for aniline substrates. Plant Physiol..

[50] Mott R.L., Steward F.C. (1972). Solute Accumulation in Plant Cells. Ann. Bot..

[51] Brunkard J.O., Xu M., Scarpin M.R., Chatterjee S., Shemyakina E.A., Goodman H.M., Zambryski P. (2020). TOR dynamically regulates plant cell–cell transport. Proc. Natl. Acad. Sci. USA.

[52] Bush D.R. (2020). Identifying the pathways that control resource allocation in higher plants. Proc. Natl. Acad. Sci. USA.

[53] Xu Q., Yin S., Ma Y., Song M., Song Y., Mu S., Li Y., Liu X., Ren Y., Gao C. (2020). Carbon export from leaves is controlled via ubiquitination and phosphorylation of sucrose transporter SUC2. Proc. Natl. Acad. Sci. USA.

[54] Chupeau M.-C., Lemoine M., Chupeau Y. (1993). Requirement of thidiazuron for healthy protoplast development to efficient tree regeneration of a hybrid poplar (Populus tremula ß p. alba). J. Plant Physiol..

[55] Li Z., Traore A., Maximova S., Guiltinan M.J. (1998). Somatic embryogenesis and plant regeneration from floral explants of cacao (Theobroma cacao L.) using thidiazuron. Vitro Cell. Dev. Biol. Plant.

[56] Wang S.Y., Ji Z.L., Sun T., Faust M. (1987). Effect of thidiazuron on abscisic acid content in apple bud relative to dormancy. Physiol. Plant..

[57] Wang S.Y., Ji Z.L., Faust M. (1987). Metabolic changes associated with bud break induced by thidiazuron. J. Plant Growth Regul..

[58] Kiba T., Takebayashi Y., Kojima M., Sakakibara H. (2019). Sugar-induced de novo cytokinin biosynthesis contributes to Arabidopsis growth under elevated CO2. Sci Rep..

[59] EI-D A.M.S.A., Salama A., Wareing P.F. (1979). Effects of Mineral Nutrition on Endogenous Cytokinins in Plants of Sunflower (Helianthus annuus L.). J. Exp. Bot..

[60] Kamada-Nobusada T., Makita N., Kojima M., Sakakibara H. (2013). Nitrogen-Dependent Regulation of De Novo Cytokinin Biosynthesis in Rice: The Role of Glutamine Metabolism as an Additional Signal. Plant. Cell Physiol..

[61] Murch S.J., Saxena P.K. (1997). Modulation of mineral and free fatty acid profiles during thidiazuron mediated somatic embryogenesis in peanuts (Arachis hypogeae L.). J. Plant Physiol..

[62] Lange B.M., Rujan T., Martin W., Croteau R. (2000). Isoprenoid biosynthesis: The evolution of two ancient and distinct pathways across genomes. Proc. Natl. Acad. Sci. USA.

[63] Mahmoud S.S., Croteau R.B. (2002). Strategies for transgenic manipulation of monoterpene biosynthesis in plants. Trends Plant Sci..

[64] Stermer B.A., Bianchini G.M., Korth K.L. (1994). Regulation of HMG-CoA reductase activity in plants. J. Lipid Res..

[65] Dewir Y.H., Nurmansyah, Naidoo Y., da Silva J.A.T. (2018). Thidiazuron-induced abnormalities in plant tissue cultures. Plant Cell Rep..

[66] Lu H., Xu P., Hu K., Xiao Q., Wen J., Yi B., Ma C., Tu J., Fu T., Shen J. (2020). Transcriptome profiling reveals cytokinin promoted callus regeneration in Brassica juncea. Plant Cell Tissue Organ Cult..

[67] Mok M.C., Martin R.C., Dobrev P.I., Vanková R., Ho P.S., Yonekura-Sakakibara K., Sakakibara H., Mok D.W.S. (2005). Topolins and Hydroxylated Thidiazuron Derivatives Are Substrates of Cytokinin O-Glucosyltransferase with Position Specificity Related to Receptor Recognition. Plant Physiol..

[68] Louie G.V., Bowman M.E., Moffitt M.C., Baiga T.J., Moore B.S., Noel J.P. (2006). Structural determinants and modulation of substrate specificity in phenylalanine-tyrosine ammonia-lyases. Chem. Biol..

[69] Erland L.A.E., Chattopadhyay A., Jones A.M.P., Saxena P.K. (2016). Melatonin in plants and plant culture systems: Variability, stability and efficient quantification. Front. Plant Sci..

[70] Skoog F., Miller C.O. (1957). Chemical regulation of growth and organ formation in plant tissues cultured in vitro. Symp. Soc. Exp. Biol..

[71] Charchoglyan A., Abrahamyan A., Fujii I., Boubakir Z., Gulder T.A.M., Kutchan T.M., Vardapetyan H., Bringmann G., Ebizuka Y., Beerhues L. (2007). Differential accumulation of hyperforin and secohyperforin in Hypericum perforatum tissue cultures. Phytochemistry.

[72] Da Silva J.A.T., Dobránszki J., Ross S. (2013). Phloroglucinol in plant tissue culture. Vitro Cell. Dev. Biol. Plant.

[73] Steward F.C., Bidwell R.G.S., Yemm E.W. (1958). Nitrogen Metabolism, Respiration, and Growth of Cultured Plant Tissue: Part I. Experimental design, techniques, and recorded data: Part II. The interpretation of specific activity data in tracer experiments: Part III. Nitrogen metabolism and respiration of carrot tissue explants as revealed by experiments with C 14-Labelled Substrates. J. Exp. Bot..

[74] Steward F.C., Kent A.E., Mapes M.O. (1967). Growth and organization in cultured cells: Sequential and synergistic effects of growth-regulating substances. Ann. N. Y. Acad. Sci..

[75] Gill R., Saxena P.K. (1992). Direct somatic embryogenesis and regeneration of plants from seedling explants of peanut (Arachis hypogaea): Promotive role of thidiazuron. Can. J. Bot..

[76] Malik K.A., Saxena P.K. (1992). Thidiazuron Induces High-Frequency Shoot Regeneration in Intact Seedlings of Pea (Pisum-Sativum), Chickpea (Cicer-Arietinum) and Lentil (Lens-Culinaris). Aust. J. Plant Physiol..

[77] Saxena P.K., Malik K.A., Gill R. (1992). Induction by thidiazuron of somatic embryogenesis in intact seedlings of peanut. Planta.

[78] Murch S.J., Campbell S.S.B., Saxena P.K. (2001). The role of serotonin and melatonin in plant morphogenesis: Regulation of auxin-induced root organogenesis in in vitro-cultured explants of st. John’s Wort (Hypericum perforatum L.). Vitro Cell. Dev. Biol. Plant.

[79] Erland L.A.E., Murch S.J., Reiter R.J., Saxena P.K. (2015). A new balancing act: The many roles of melatonin and serotonin in plant growth and development. Plant Signal. Behav..

[80] Erland L.A.E., Shukla M.R., Singh A.S., Murch S.J., Saxena P.K. (2018). Melatonin and serotonin: Mediators in the symphony of plant morphogenesis. J. Pineal Res..

[81] Chattopadhyay A., Erland L.A.E., Jones A.M.P., Saxena P.K. (2018). Indoleamines and phenylpropanoids modify development in the bryophyte Plagiomnium cuspidatum. Vitro Cell. Dev. Biol. Plant.

[82] Street H., Sharp W.R., Larsen R.P.O., Paddock E.F., Raghavan V. (1979). Embryogenesis and chemically induced organogenesis. Plant and Cell Tissue Culture: Principles and Application.

