# Supplementary material for: The Morphoregulatory Role of Thidiazuron: Metabolomics-Guided Hypothesis Generation for Mechanisms of Activity
Source: Biomolecules. 2020 Aug 28;10(9):1253. doi: 10.3390/biom10091253 (PMC7564436; doi:10.3390/biom10091253)
Supplement: Supplementary file 1 [file biomolecules-10-01253-s001.zip › Supplementary Files/Supplementary-Tables_rev1.docx]

**Supplementary Tables**

**Supplementary Table 1.** Standard Operating Protocol methods of extraction and ionization of metabolites as described by Aharoni et al. 2002.

| **Mode** | **Ionization Mode** | **Chemical Class** |
| --- | --- | --- |
| **Mode 11** | Positive Ion Electrospray | Highly polar compounds including quaternary amines, zwitterions and aminosugars etc. |
| **Mode 12** | Negative Ion Electrospray | Highly polar compounds including sugars, phosphates etc. |
| **Mode 41** | Positive Ion Electrospray | Organic bases |
| **Mode 32** | Negative Ion Electrospray | Organic acids |
| **Mode 31** | Positive Ion Electrospray | Polar neutrals |
| **Mode 33** | Positive Atmospheric Pressure Chemical | Non-polar neutrals |

**Supplementary Table 2.** Summary of query masses for hormonomics analysis. 5HT, serotonin; ABA, abscisic acid; IAA, indole-3-acetic acid; JA, jasmonic acid; MEL, melatonin; Trp, tryptophan;

| **Class** | **Metabolite Name** | **M** | **M+H** |
| --- | --- | --- | --- |
| 5-HT conjugates | 5HT-Alanine | 229.116673 | 230.124498 |
| 5-HT conjugates | 5HT-Arginine | 314.180673 | 315.188498 |
| 5-HT conjugates | 5HT-Asaparagine | 272.122493 | 273.130318 |
| 5-HT conjugates | 5HT-Aspartate | 273.106503 | 274.114328 |
| 5-HT conjugates | 5HT-Cysteine | 261.088753 | 262.096578 |
| 5-HT conjugates | 5HT-Glutamate | 287.122153 | 288.129978 |
| 5-HT conjugates | 5HT-Glutamine | 286.138143 | 287.145968 |
| 5-HT conjugates | 5HT-Glycine | 215.101023 | 216.108848 |
| 5-HT conjugates | 5HT-Histidine | 295.138473 | 296.146298 |
| 5-HT conjugates | 5HT-Isoleucine/Leucine | 271.163623 | 272.171448 |
| 5-HT conjugates | 5HT-Lysine | 286.174523 | 287.182348 |
| 5-HT conjugates | 5HT-Methionine | 289.120053 | 290.127878 |
| 5-HT conjugates | 5HT-Phenyalanine | 305.147973 | 306.155798 |
| 5-HT conjugates | 5HT-Proline | 255.132323 | 256.140148 |
| 5-HT conjugates | 5HT-Serine | 245.111593 | 246.119418 |
| 5-HT conjugates | 5HT-Threonine | 259.127243 | 260.135068 |
| 5-HT conjugates | 5HT-Tryptophan | 344.158873 | 345.166698 |
| 5-HT conjugates | 5HT-Tyrosine | 321.142893 | 322.150718 |
| 5-HT conjugates | 5HT-Valine | 257.147973 | 258.155798 |
| ABA | 7/9-hydroxy-ABA | 281.1389 | 282.146725 |
| ABA | Abscisic acid | 264.136159 | 265.143984 |
| ABA | dihydrophaseic acid | 282.146724 | 283.154549 |
| ABA | neophaseic acid | 279.123249 | 280.131074 |
| ABA | phaseic acid | 280.131074 | 281.138899 |
| Auxins | 2-oxo-indole-3-acetic acid | 191.18336 | 192.191185 |
| Auxins | anthranilic acid | 137.047678 | 138.055503 |
| Auxins | IAA alanine | 246.100442 | 247.108267 |
| Auxins | IAA aspartate | 450.127445 | 451.13527 |
| Auxins | IAA glutamate | 464.143095 | 465.15092 |
| Auxins | IAA glycine | 232.23528 | 233.243105 |
| Auxins | IAA leucine | 288.147393 | 289.155218 |
| Auxins | IAA phenylalanine | 322.131742 | 323.139567 |
| Auxins | IAA valine | 289.118832 | 290.126657 |
| Auxins | indol-3-acetamide | 524.269988 | 525.277813 |
| Auxins | indol-3-acetic acid | 175.063329 | 176.071154 |
| Auxins | indole-3-acetonitrile | 156.068748 | 157.076573 |
| Auxins | indole-3-butyric acid | 203.094629 | 204.102454 |
| Brassinosteroids | 24-epicastasterone | 464.350175 | 465.358 |
| Brassinosteroids | 28-norcastasterone | 450.334525 | 451.34235 |
| Brassinosteroids | brassinolide | 480.345089 | 481.352914 |
| Brassinosteroids | castasterone | 464.350175 | 465.358 |
| Brassinosteroids | dolicholide | 478.329439 | 479.337264 |
| Brassinosteroids | dolichosterone | 462.334525 | 463.34235 |
| Brassinosteroids | homobrassinolide | 494.360739 | 495.368564 |
| Brassinosteroids | homocastasterone | 478.365825 | 479.37365 |
| Brassinosteroids | homodolicholide | 492.345089 | 493.352914 |
| Brassinosteroids | homodolichosterone | 476.350175 | 477.358 |
| Brassinosteroids | norbrassinolide | 466.329439 | 467.337264 |
| Brassinosteroids | teasterone | 448.35526 | 449.363085 |
| Brassinosteroids | typhasterol | 448.35526 | 449.363085 |
| Catecholamines | betalamic acid | 211.048072 | 212.055897 |
| Catecholamines | cyclo-dopa | 195.053158 | 196.060983 |
| Catecholamines | cyclo-dopa-5-O-glucoside | 357.105981 | 358.113806 |
| Catecholamines | dopamine | 153.078979 | 154.086804 |
| Catecholamines | dopaquinone | 195.053158 | 196.060983 |
| Catecholamines | Epinephrine | 183.089543 | 184.097368 |
| Catecholamines | levodopa | 197.068808 | 198.076633 |
| Catecholamines | norepinephrine | 169.073893 | 170.081718 |
| Catecholamines | tyramine | 137.084064 | 138.091889 |
| Catecholamines | Tyrosine | 181.073893 | 182.081718 |
| Cytokinins | 2-methylthio-cis-zeatin | 265.099732 | 266.107557 |
| Cytokinins | 2-methylthio-cis-zeatin riboside | 397.14199 | 398.149815 |
| Cytokinins | 2-methylthio-isopentenyladenine | 249.104817 | 250.112642 |
| Cytokinins | 2-methylthio-isopentenyladenosine | 381.147077 | 382.154902 |
| Cytokinins | dihydrozeatin | 221.12766 | 222.135485 |
| Cytokinins | dihydrozeatin riboside-O-glucoside | 515.222745 | 516.23057 |
| Cytokinins | dihydrozeatin-(7/9)-glucoside | 383.180484 | 384.188309 |
| Cytokinins | kinetin | 215.08071 | 216.088535 |
| Cytokinins | kinetin-9-glucoside | 377.133535 | 378.14136 |
| Cytokinins | kinetin-riboside | 347.12297 | 348.130795 |
| Cytokinins | N6-benzyladenine | 225.101445 | 226.10927 |
| Cytokinins | N6-benzyladenine riboside | 357.143704 | 358.151529 |
| Cytokinins | N6-benzyladenine-(7/9)-glucoside | 519.196529 | 520.204354 |
| Cytokinins | N6-isopentenyladenine | 203.117095 | 204.12492 |
| Cytokinins | N6-isopentenyladenine-(7/9)-glucoside | 365.169922 | 366.177747 |
| Cytokinins | N6-isopentenyladenosine | 415.125685 | 416.13351 |
| Cytokinins | para/meta/ortho-topolin | 241.09636 | 242.104185 |
| Cytokinins | para/meta/ortho-topolin riboside | 373.138619 | 374.146444 |
| Cytokinins | para/meta/ortho-topolin-9-glucoside | 419.144098 | 420.151923 |
| Cytokinins | zeatin | 219.11201 | 220.119835 |
| Cytokinins | zeatin riboside | 351.154269 | 352.162094 |
| Cytokinins | zeatin riboside-O-glucoside | 513.207092 | 514.214917 |
| Cytokinins | zeatin-(7/9/O)-glucoside | 381.164833 | 382.172658 |
| Gibberellins | Gibberellin A1/A29/A34 | 348.157288 | 349.165113 |
| Gibberellins | Gibberellin A15 | 344.198759 | 345.206584 |
| Gibberellins | Gibberellin A19 | 362.172939 | 363.180764 |
| Gibberellins | Gibberellin A24/A44 | 346.178024 | 347.185849 |
| Gibberellins | Gibberellin A3/A6 | 346.141638 | 347.149463 |
| Gibberellins | Gibberellin A4/A51 | 332.162374 | 333.170199 |
| Gibberellins | Gibberellin A5 | 330.146724 | 331.154549 |
| Gibberellins | Gibberellin A53 | 348.193674 | 349.201499 |
| Gibberellins | Gibberellin A8 | 364.152203 | 365.160028 |
| Indoleamine | bufotenine | 204.126263 | 205.133539 |
| Indoleamine | caffeoyl serotonin | 338.126657 | 339.133933 |
| Indoleamine | cinnamoyl serotonin | 306.136828 | 307.144104 |
| Indoleamine | cyclic-melatonin | 230.105528 | 231.113353 |
| Indoleamine | feruloyl serotonin | 352.142307 | 353.149583 |
| Indoleamine | N-3,4-dimethoxy cinnamoyl serotonin | 366.157957 | 367.165233 |
| Indoleamine | N-coumaroylserotonin | 322.131742 | 323.139018 |
| Indoleamine | N-P-Methoxy cinnamoyl serotonin | 336.147393 | 337.154669 |
| Indoleamine | sinapoyl serotonin | 382.152872 | 383.160148 |
| Indoleamines | (2/4/6/7)-hydroxymelatonin | 248.116089 | 249.123914 |
| Indoleamines | 5-Methoxy-1H-indole-3-carbaldehyde | 175.063329 | 176.071154 |
| Indoleamines | 5-methoxyindole-3 acetic acid | 205.073893 | 206.081718 |
| Indoleamines | 5-methoxytryptamine; O-methylserotonin | 190.110613 | 191.118438 |
| Indoleamines | 5-methoxytryptophol | 191.094629 | 192.102454 |
| Indoleamines | cyclic (3)-hydroxymelatonin | 246.100442 | 247.108267 |
| Indoleamines | kynuramine | 164.094963 | 165.102788 |
| Indoleamines | melatonin | 232.121178 | 233.129003 |
| Indoleamines | N1-acetyl-5-methoxykynuramine | 236.116092 | 237.123917 |
| Indoleamines | N6-N2-formyl-5-methoxykynuramine | 264.111007 | 265.118832 |
| Indoleamines | serotonin | 176.094963 | 177.102788 |
| Indoleamines | tryptamine | 160.100048 | 161.107873 |
| Indoleamines | tryptophan | 204.089878 | 205.097703 |
| Jasmonates | 12-hydroxy-jasmonic acid | 388.173332 | 389.181157 |
| Jasmonates | 3-oxo-2-(2-(Z)-pentenyl)cyclopentane-1-butyric acid | 238.156895 | 239.16472 |
| Jasmonates | 3-oxo-2-(2-(Z)-pentenyl)cyclopentane-1-hexanoic acid | 266.188195 | 267.19602 |
| Jasmonates | 9,10-dihydrojasmonic acid | 212.141244 | 213.149069 |
| Jasmonates | cis-12-oxo-phytodienoic acid | 292.203845 | 293.21167 |
| Jasmonates | dinor-12-oxo-phytodienoic acid | 264.172747 | 265.180572 |
| Jasmonates | JA-isoleucine | 322.201833 | 323.209658 |
| Jasmonates | JA-pheylalanine | 360.181099 | 361.188924 |
| Jasmonates | JA-tryptophan | 399.191998 | 400.199823 |
| Jasmonates | JA-valine | 312.181099 | 313.188924 |
| Jasmonates | jasmonic acid | 210.125594 | 211.133419 |
| Mel Conjugates | caffeoylMEL | 394.152872 | 395.160697 |
| Mel Conjugates | cinnamoylMEL | 362.163043 | 363.170868 |
| Mel Conjugates | feruloylMEL | 408.168522 | 409.176347 |
| Mel Conjugates | MEL-Ala | 285.142888 | 286.150713 |
| Mel Conjugates | MEL-Arg | 370.206888 | 371.214713 |
| Mel Conjugates | MEL-Asn | 328.148708 | 329.156533 |
| Mel Conjugates | MEL-Asp | 329.132718 | 330.140543 |
| Mel Conjugates | MEL-Cys | 317.114968 | 318.122793 |
| Mel Conjugates | MEL-Gln | 342.164358 | 343.172183 |
| Mel Conjugates | MEL-Glu | 343.148368 | 344.156193 |
| Mel Conjugates | MEL-Gly | 271.127238 | 272.135063 |
| Mel Conjugates | MEL-His | 351.164688 | 352.172513 |
| Mel Conjugates | MEL-Ile | 327.189838 | 328.197663 |
| Mel Conjugates | MEL-Leu | 327.189838 | 328.197663 |
| Mel Conjugates | MEL-Lys | 342.200738 | 343.208563 |
| Mel Conjugates | MEL-Met | 345.146268 | 346.154093 |
| Mel Conjugates | MEL-Phe | 361.174188 | 362.182013 |
| Mel Conjugates | MEL-Pro | 311.158538 | 312.166363 |
| Mel Conjugates | MEL-Ser | 301.137808 | 302.145633 |
| Mel Conjugates | MEL-Thr | 315.153458 | 316.161283 |
| Mel Conjugates | MEL-Trp | 400.185088 | 401.192913 |
| Mel Conjugates | MEL-Tyr | 377.169108 | 378.176933 |
| Mel Conjugates | MEL-Val | 313.174188 | 314.182013 |
| Mel Conjugates | N-coumaroylMEL | 378.157957 | 379.165782 |
| Mel Conjugates | sinapoylMEL | 438.179087 | 439.186912 |
| Mitragynine |  | 398.220557 | 399.228382 |
| SA | Salicyclic acid | 138.031694 | 139.039519 |
| Trp metabolism | 2-amino-3-carboxymuconic acid¬†semialdehyde | 185.032425 | 186.04025 |
| Trp metabolism | 2-Aminomuconic semialdehyde | 141.042593 | 142.050418 |
| Trp metabolism | 3-hydroxyanthranilate | 152.035324 | 153.043149 |
| Trp metabolism | 3-hydroxykynurenine | 224.079712 | 225.087537 |
| Trp metabolism | 5-HTP | 220.084793 | 221.092618 |
| Trp metabolism | aminomuconate | 156.029683 | 157.037508 |
| Trp metabolism | aniline | 93.057846 | 94.065671 |
| Trp metabolism | anthranilate | 136.040405 | 137.04823 |
| Trp metabolism | formylanthranilate | 164.034768 | 165.042593 |
| Trp metabolism | formylkynurenine | 236.079712 | 237.087537 |
| Trp metabolism | kynurenine | 208.084793 | 209.092618 |
| Trp metabolism | nicotinic acid mononucleotide | 336.047882 | 337.055707 |
| Trp metabolism | picolinate | 123.032028 | 124.039853 |
| Trp metabolism | quinolinate | 172.040405 | 173.04823 |
| Trp metabolism | quinolinic acid | 167.021858 | 168.029683 |

**Supplementary Table 3.** Summary of metabolomic database.

|  | **Control** | **Low TDZ** | **High TDZ** |
| --- | --- | --- | --- |
| Total Predicted Metabolites in the Database | 18602 | | |
| Total Predicted Metabolites | 8591 | 8791 | 7692 |
| Common to All | 2236 | | |
| Common to Control and Low TDZ | 3042 | | |
| Common to Control and High TDZ | 2870 | | |
| Common to Control and Low TDZ; Absent High TDZ | 806 | |  |
| Common to Control and High TDZ; Absent Low TDZ | 634 |  | 634 |
| Common to TDZ treatments; Absent Control |  | 1412 | |
| Absent Control; Increasing with Increasing TDZ | 312 | | |
| Highest in Control; Decreasing with Increasing TDZ | 1327 | | |
| Highest in High TDZ; Absent in Control and Low TDZ |  |  | 4347 |
| Highest in Low TDZ; Absent in Control and High TDZ |  | 5863 |  |
