# Supplementary figures and images for: The Morphoregulatory Role of Thidiazuron: Metabolomics-Guided Hypothesis Generation for Mechanisms of Activity
Source: Biomolecules. 2020 Aug 28;10(9):1253. doi: 10.3390/biom10091253 (PMC7564436; doi:10.3390/biom10091253)

Figure S1:

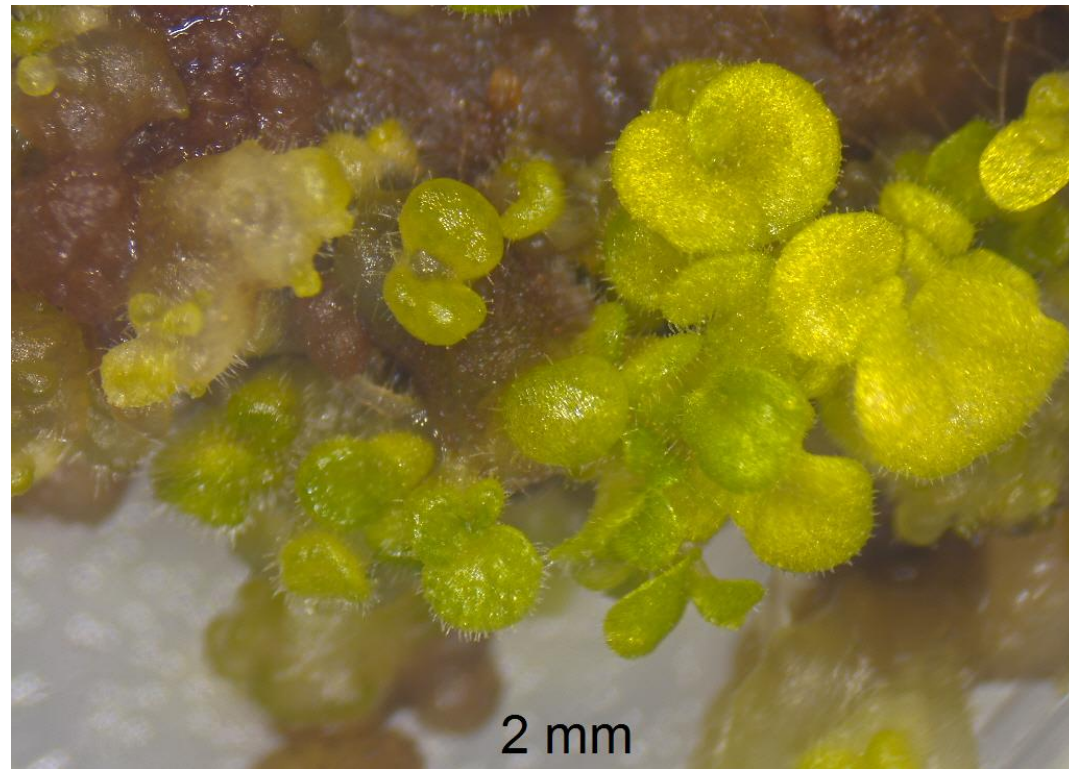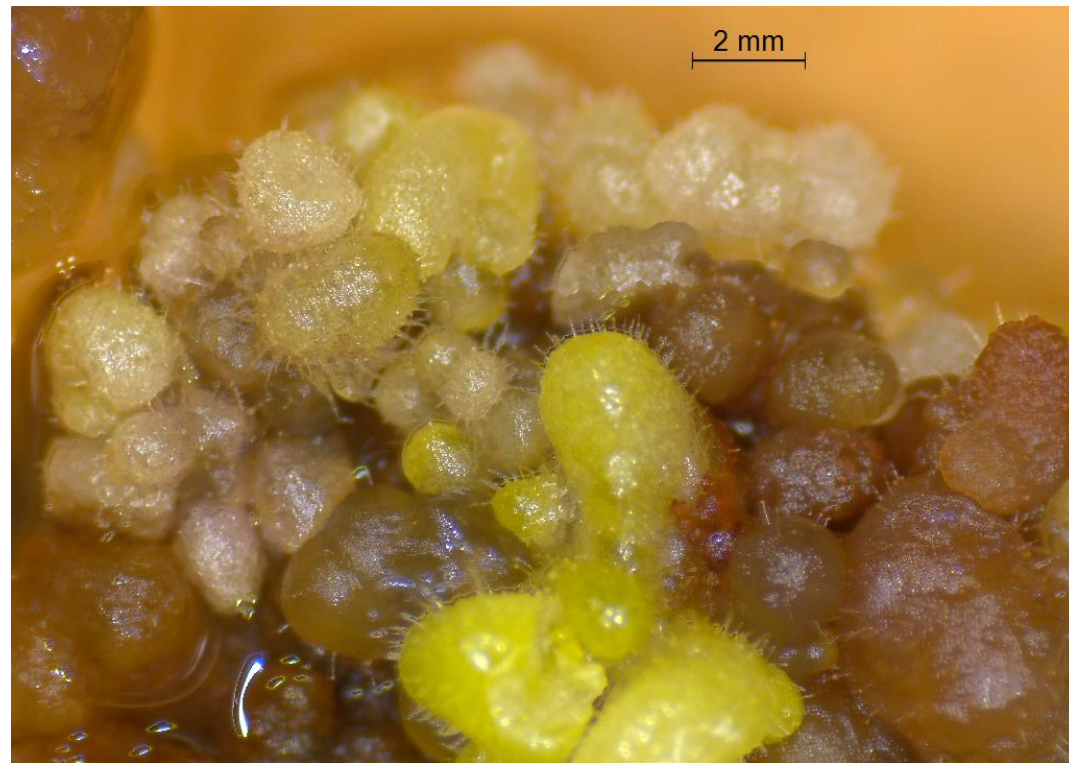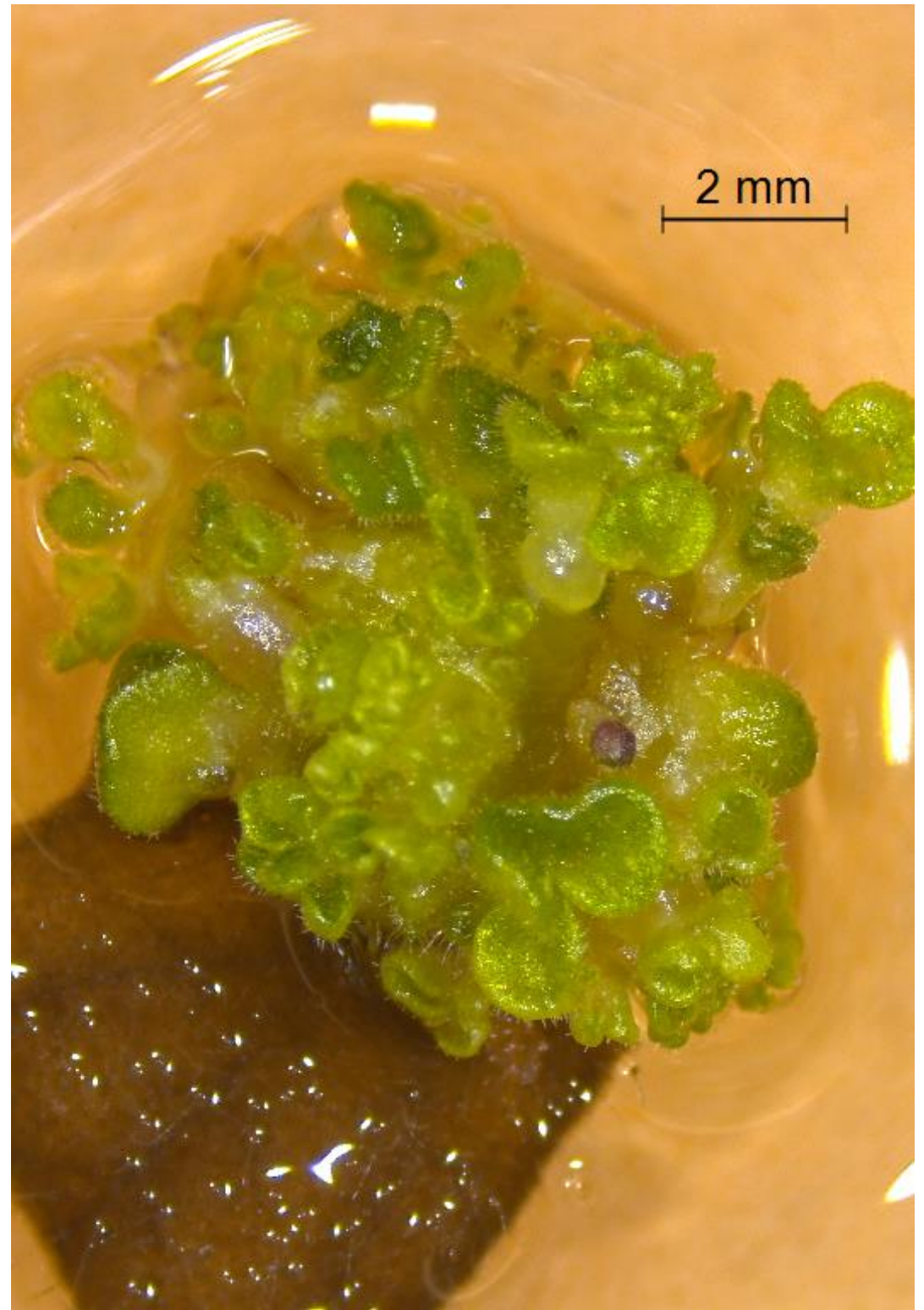

Figure S2:

**a**

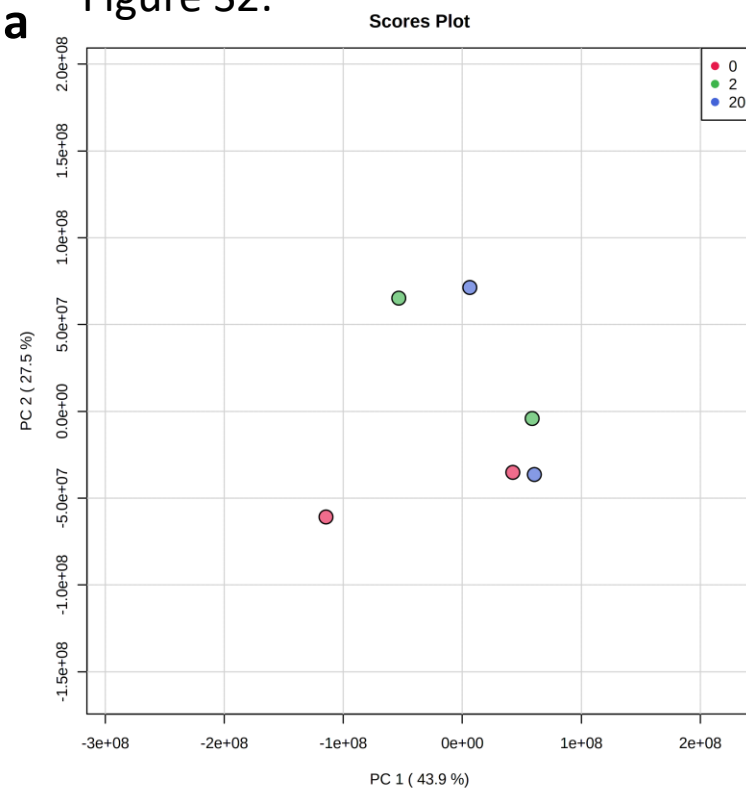

**b**

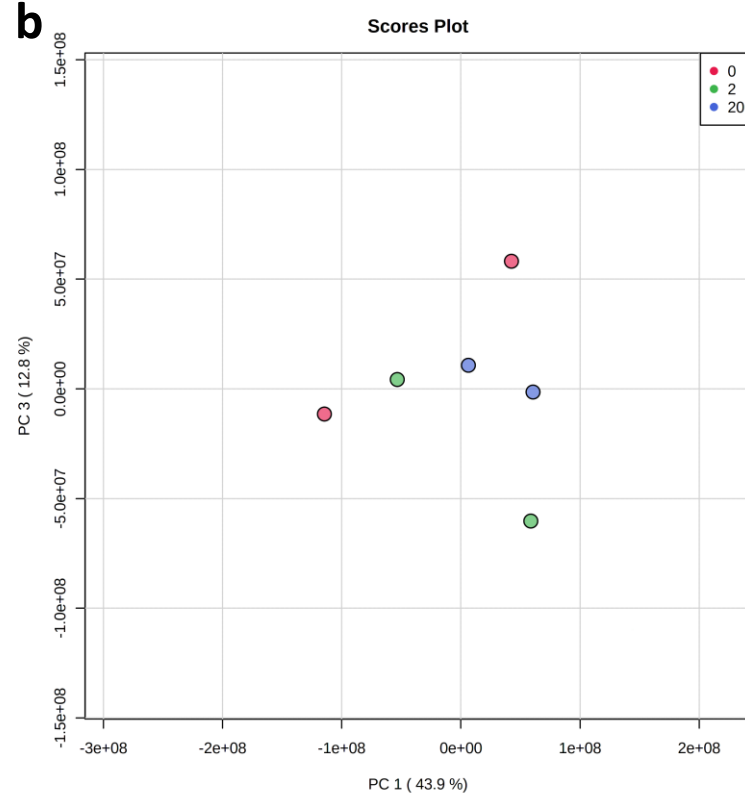

**c**

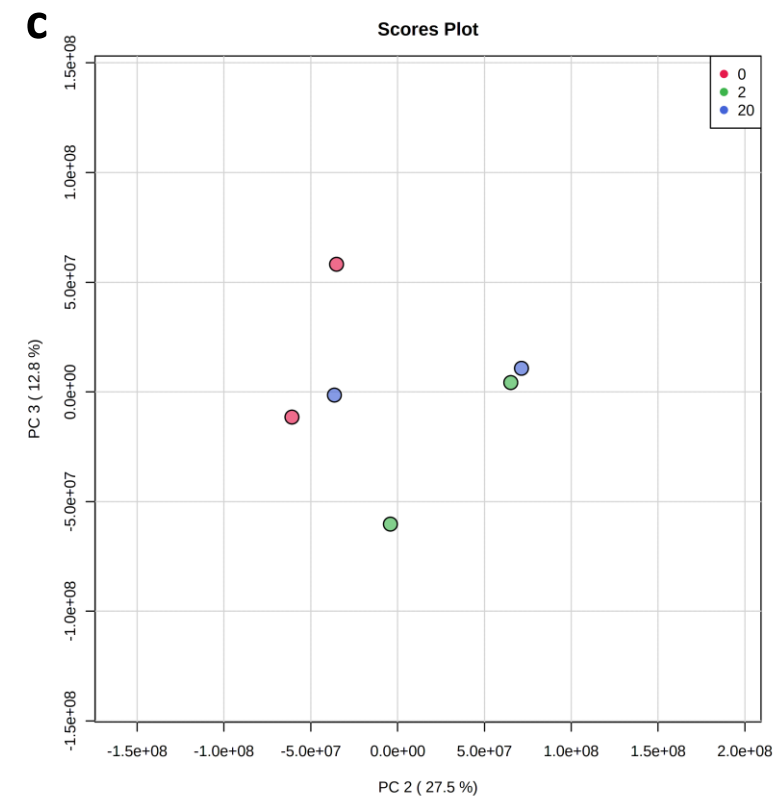

**d**

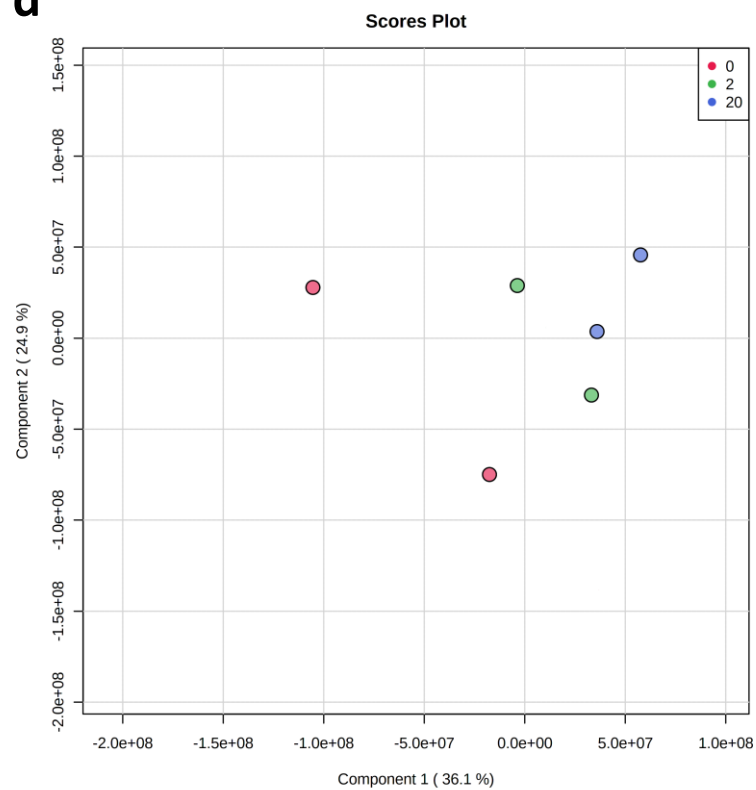

**e**

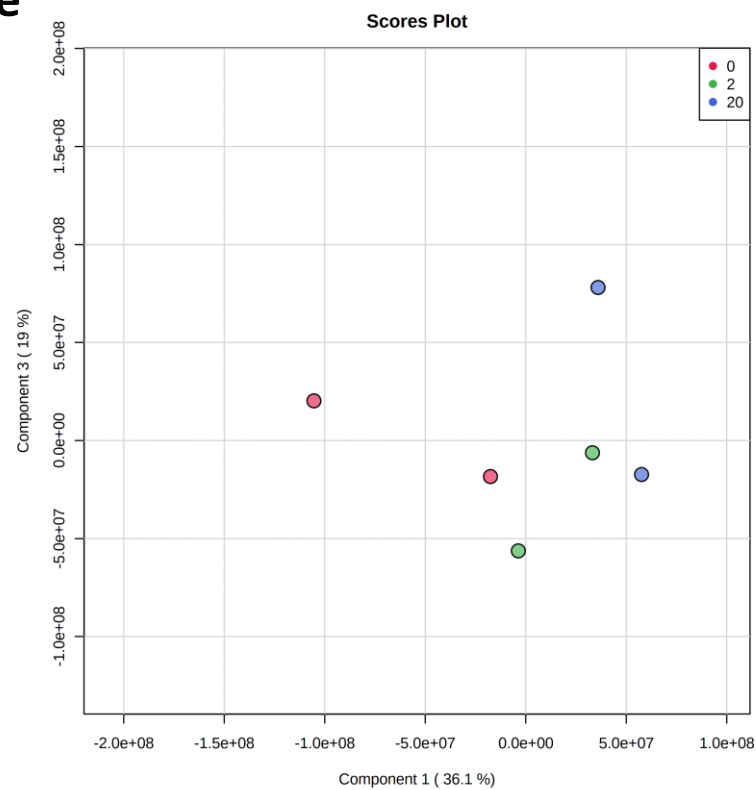

**f**

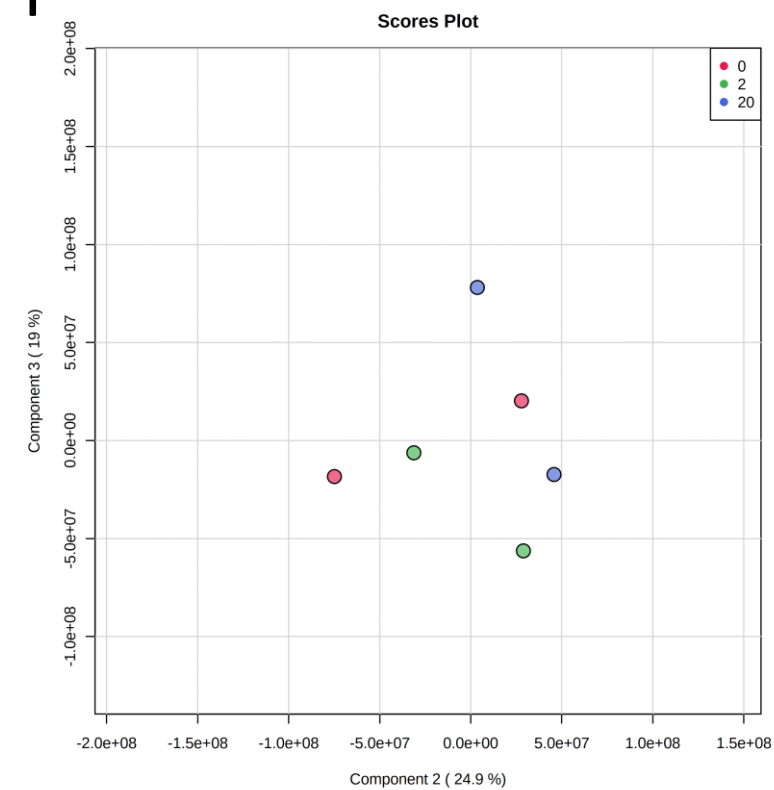

Figure S3:

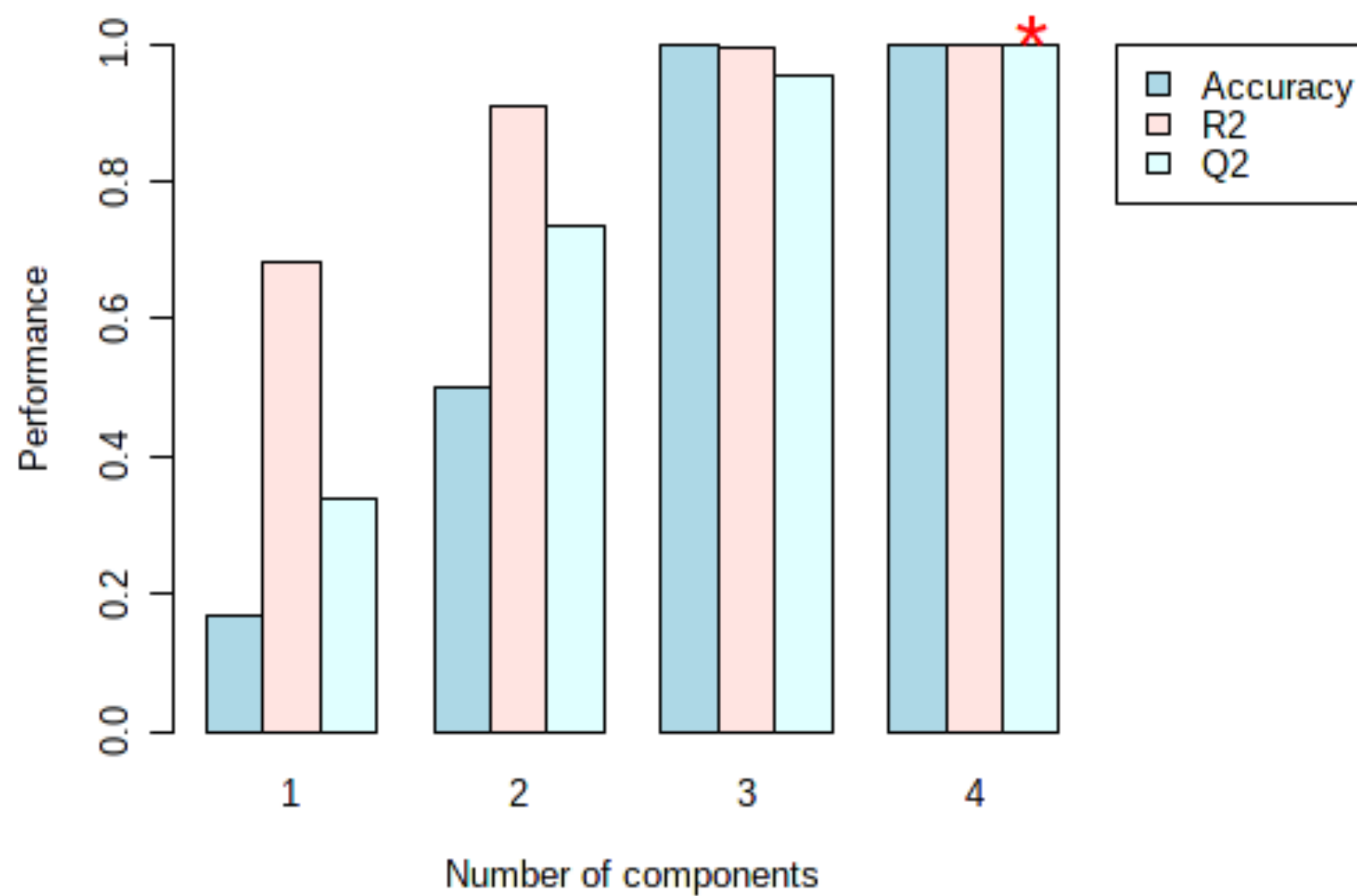

Figure S4:

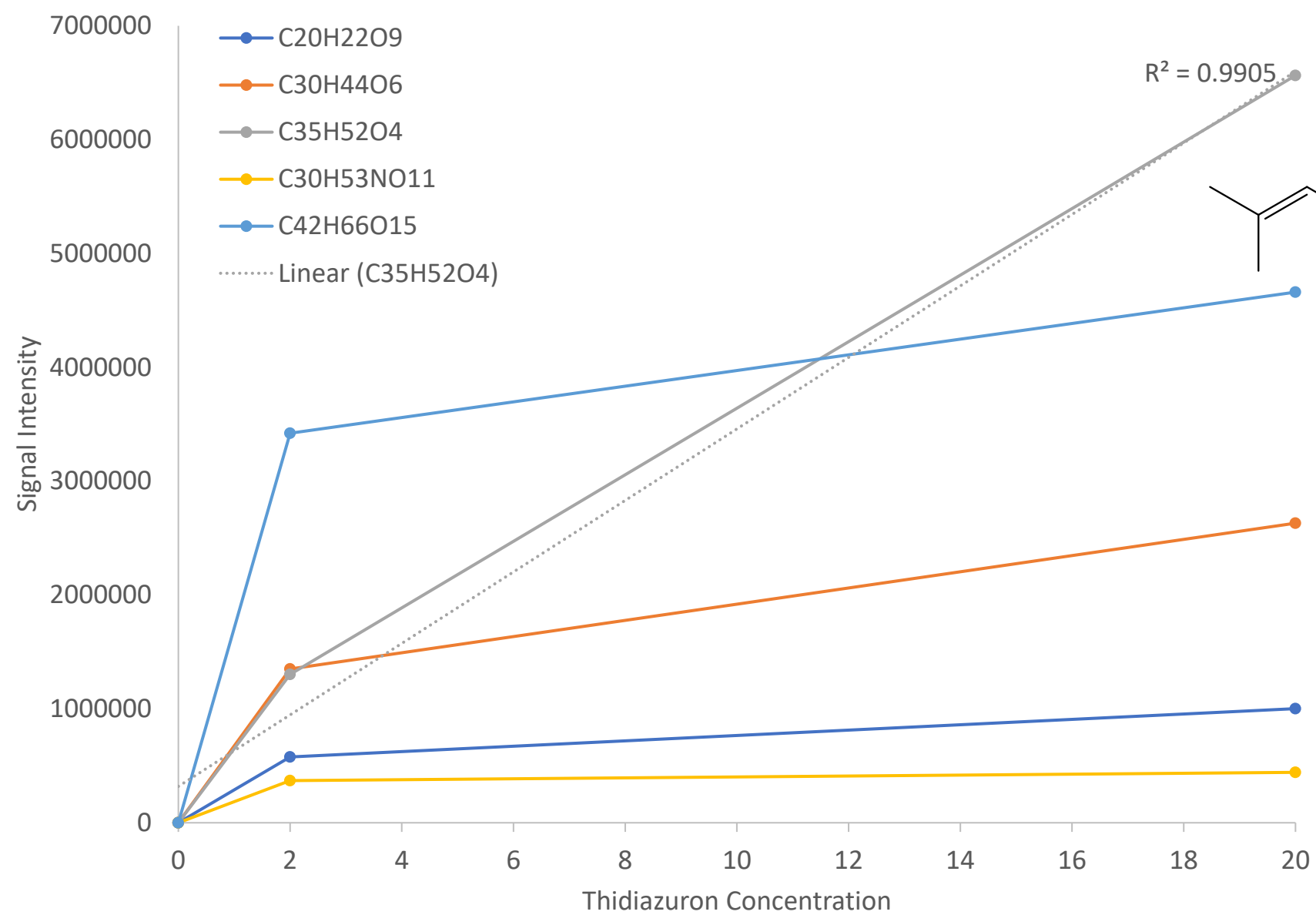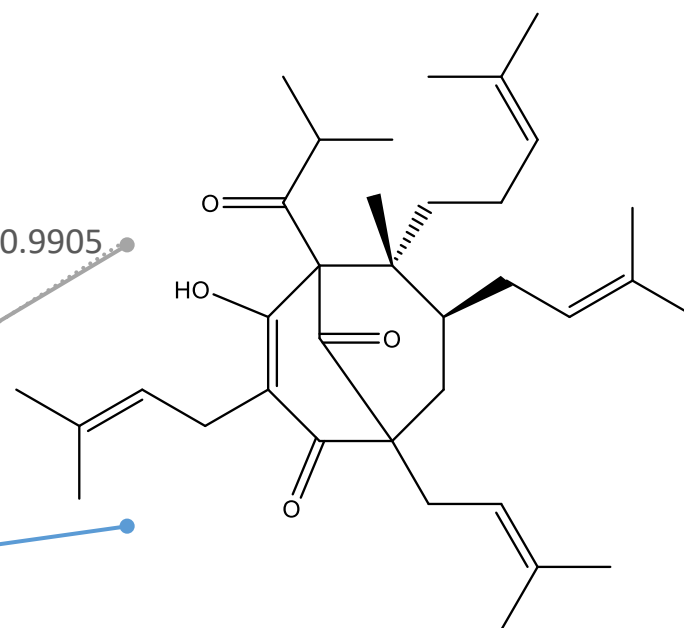

Figure S5:

A

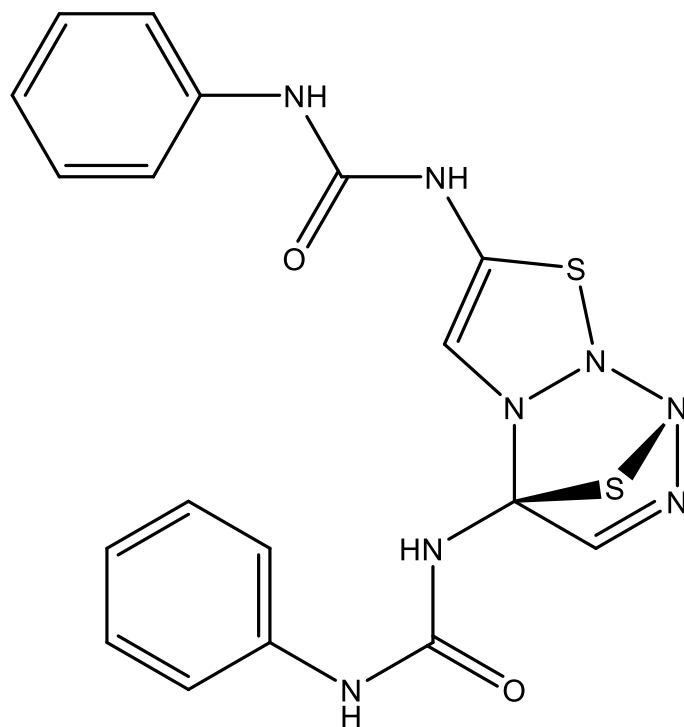

B

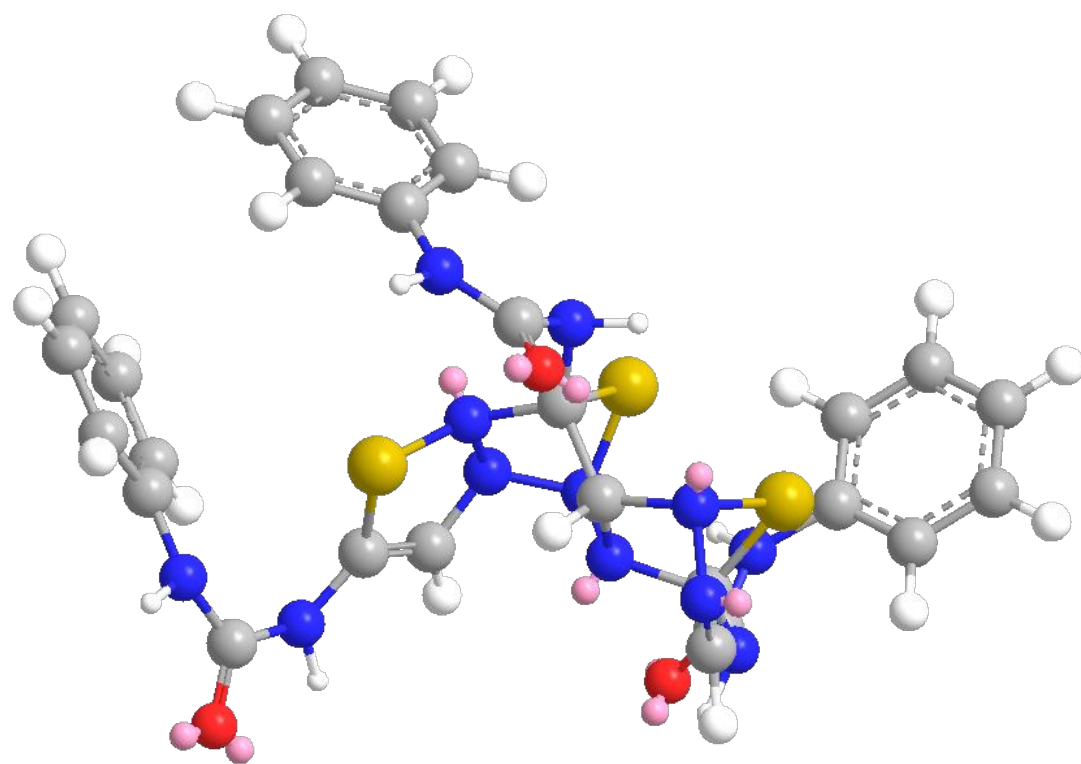

C

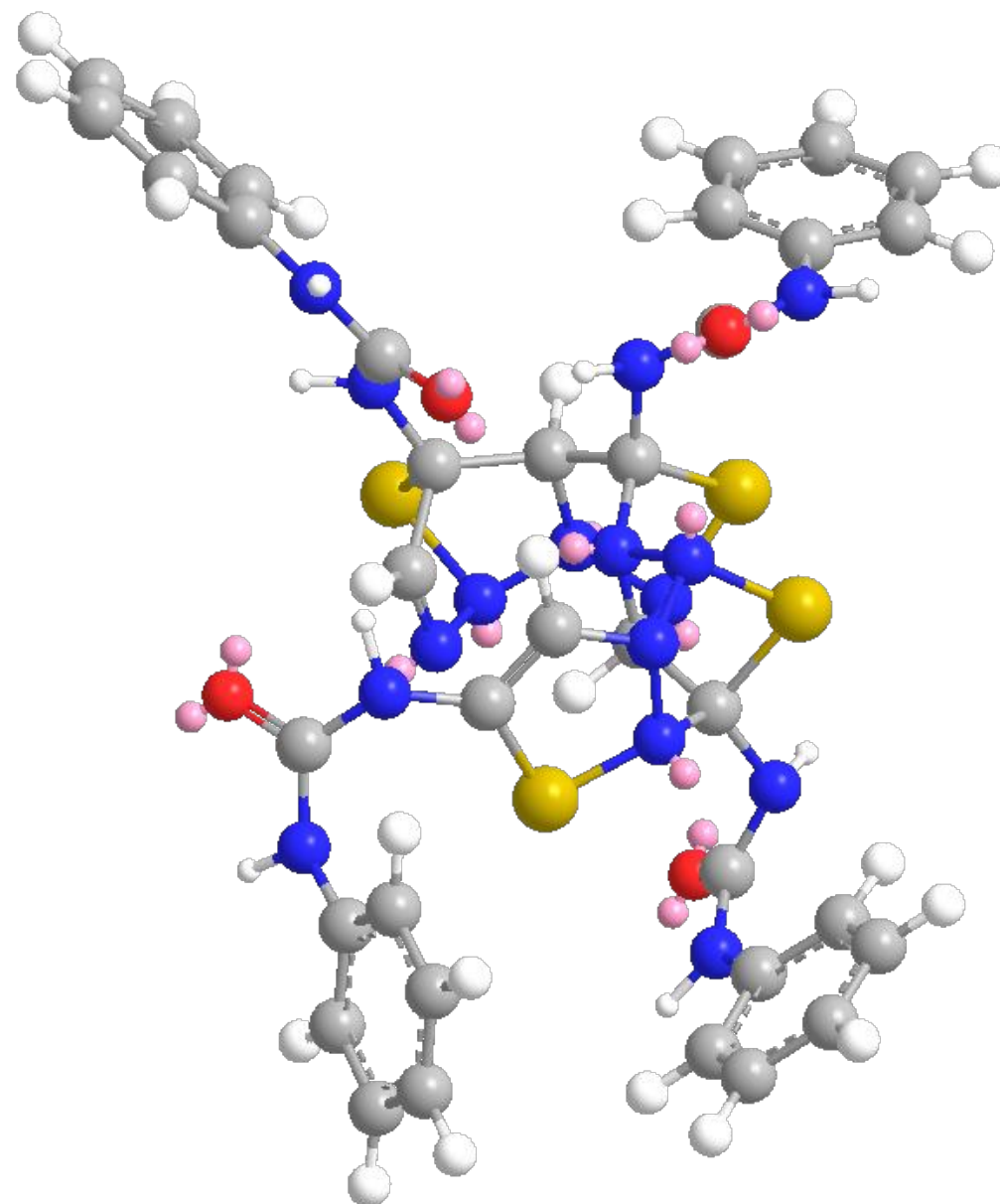

Supplement: Supplementary file 1 [file biomolecules-10-01253-s001.zip › Supplementary Files/Supplementary Figures.pdf]
